# *Drosophila* p38 MAPK interacts with BAG‐3/starvin to regulate age‐dependent protein homeostasis

**DOI:** 10.1111/acel.13481

**Published:** 2021-10-21

**Authors:** Sarah M. Ryan, Michael Almassey, Amelia M. Burch, Gia Ngo, Julia M. Martin, David Myers, Devin Compton, Shira Archie, Megan Cross, Lauren Naeger, Ashley Salzman, Alyssa Virola‐Iarussi, Scott A. Barbee, Nathan T. Mortimer, Subhabrata Sanyal, Alysia D. Vrailas‐Mortimer

**Affiliations:** ^1^ Department of Biological Sciences University of Denver Denver CO USA; ^2^ School of Biological Sciences Illinois State University Normal IL USA; ^3^ Department of Cell Biology Emory University Atlanta GA USA; ^4^ Calico San Francisco CA USA

**Keywords:** aging, BAG‐3/starvin, Lamin, p38 MAPK, protein aggregation

## Abstract

As organisms age, they often accumulate protein aggregates that are thought to be toxic, potentially leading to age‐related diseases. This accumulation of protein aggregates is partially attributed to a failure to maintain protein homeostasis. A variety of genetic factors have been linked to longevity, but how these factors also contribute to protein homeostasis is not completely understood. In order to understand the relationship between aging and protein aggregation, we tested how a gene that regulates lifespan and age‐dependent locomotor behaviors, p38 MAPK (p38Kb), influences protein homeostasis as an organism ages. We find that p38Kb regulates age‐dependent protein aggregation through an interaction with starvin, a regulator of muscle protein homeostasis. Furthermore, we have identified Lamin as an age‐dependent target of p38Kb and starvin.

## Graphical abstract

We find that p38 MAPK (p38Kb) regulates age‐dependent protein homeostasis through an interaction with the BAG protein, starvin. In addition, we find that Lamin Dm0, a homologue of the aging gene Lamin A/C, is a target of p38Kb and stv for degradation during aging.

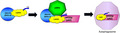

## 1 INTRODUCTION

Protein turnover is critical for maintaining tissue health as many proteins become damaged or misfolded during normal tissue functions. Therefore, the cell utilizes a variety of protein quality control mechanisms to refold or degrade these damaged proteins, including the ubiquitin proteasome system and autophagy. During aging, protein quality control mechanisms become less efficient leading to the accumulation of damaged or misfolded proteins that begin to form protein aggregates (Taylor & Dillin, 2011). It has been hypothesized that these aggregates are toxic and may lead to the deleterious phenotypes associated with normal aging, such as impaired tissue function (Taylor & Dillin, 2011). Furthermore, decreased protein aggregation has been associated with longevity. For example, over‐expression of Foxo leads to an increased lifespan but also a concordant decrease in protein aggregation in *C*. *elegans*, *Drosophila*, and mice (Ben‐Zvi et al., 2009; Cohen et al., 2006; Demontis & Perrimon, 2010; Morley et al., 2002; Palazzolo et al., 2009), suggesting that lifespan and protein aggregation are tightly linked processes. However, the molecular mechanisms that underlie the relationship between aging and protein homeostasis have not been fully characterized.

One pathway that has been linked to both aging and protein homeostasis is the stress response p38 MAPK (p38K) pathway. In mammals, there are four p38K genes (α, β, γ, and δ), and p38Kα has been linked to both the inhibition (Schnöder et al., 2016; Webber & Tooze, 2010) and induction (Liu et al., 2009; Younce & Kolattukudy, 2010) of autophagy, including in response to oxidative stress (Duan et al., 2011; Zhuang et al., 2016). In addition, p38Kα has been linked to regulating autophagy in cellular senescence (Henson et al., 2014; Luo et al., 2011; Rudolf et al., 2014). However, how p38K signaling may contribute to protein homeostasis in response to natural aging is not well understood. The fruit fly *Drosophila melanogaster* has two canonical p38K genes (p38Ka and p38Kb) that are partially redundant to each other (Craig et al., 2004; Davis et al., 2008; Ryan et al., 2020; Vrailas‐Mortimer et al., 2011), but also have distinct functions. For example, p38Ka plays a role in cardiac function (Na et al., 2013), while p38Kb regulates circadian rhythm, oxidative stress response, and aging (Ryan et al., 2020; Vrailas‐Mortimer et al., 2011, 2012, 2014). We have previously found that over‐expression of p38Kb leads to increased lifespan while loss of p38Kb results in a short lifespan and age‐dependent locomotor behavior defects (Vrailas‐Mortimer et al., 2011). Furthermore, we found that p38Kb regulates the oxidative stress response (Ryan et al., 2020; Vrailas‐Mortimer et al., 2011, 2012) and mediates lifespan through regulation of the expression of the antioxidant enzyme SOD2 (MnSOD) (Vrailas‐Mortimer et al., 2011). In addition, oxidatively damaged proteins accumulate in the muscle of p38Kb mutants with age (Vrailas‐Mortimer et al., 2011), and loss of p38Kb leads to increased poly‐ubiquitination of insoluble proteins and alterations in oxidative stress dependent translation (Belozerov et al., 2014), suggesting that these oxidatively damaged proteins may be aggregating in p38Kb mutants. Furthermore, p38Kb has been shown in a *Drosophila* cell culture system to pull down with one of the fly HspB8 homologues CG14207 (Guruharsha et al., 2011), which plays a role in the muscle by regulating protein homeostasis in the adult flight muscle as a part of a protein quality control mechanism called BAG‐3 Mediated Selective Autophagy pathway in mammalian systems or the Chaperone‐Assisted Selective Autophagy (CASA) complex in flies (Arndt et al., 2010; Stürner & Behl, 2017). The CASA complex also includes the chaperone Hsc70 and the co‐chaperone BAG‐3 (starvin (stv) in flies). BAG‐3/stv binds to specific damaged or misfolded protein substrates and brings them to the CASA complex where it binds to Hsc70 and HspB8. Those substrates that cannot be refolded by the complex are poly‐ubiquitinated and targeted to the autophagosome through a handover between BAG‐3/stv and the autophagy adaptor protein p62 (ref(2)p in flies), and subsequently degraded through the autophagosome‐lysosome system (Behl, 2011; Carra et al., 2008; Gamerdinger et al., 2009; Kettern et al., 2010; Min et al., 2008; Selcen et al., 2009; Terman et al., 2007). Furthermore, stv has been shown to play a role in regulating protein homeostasis in fly muscle (Arndt et al., 2010; Brooks et al., 2020).

Here, we report that p38Kb regulates age‐dependent muscle protein homeostasis through an interaction with stv. We find that p38Kb acts in cooperation with BAG‐3/stv and p62/ref(2)p in targeting damaged or misfolded proteins for degradation. This interaction is not only important for maintaining protein homeostasis but also for lifespan extension. In addition, we find that Lamin Dm_0_, a *Drosophila* homologue of the Hutchinson‐Gilford progeria protein, Lamin A/C, is a target for p38Kb and stv mediated protein turnover, suggesting that the p38Kb aging phenotypes may be a result of impaired Lamin degradation.

## 2 RESULTS

### 2.1 p38Kb regulates age‐dependent protein homeostasis

**FIGURE 1:**
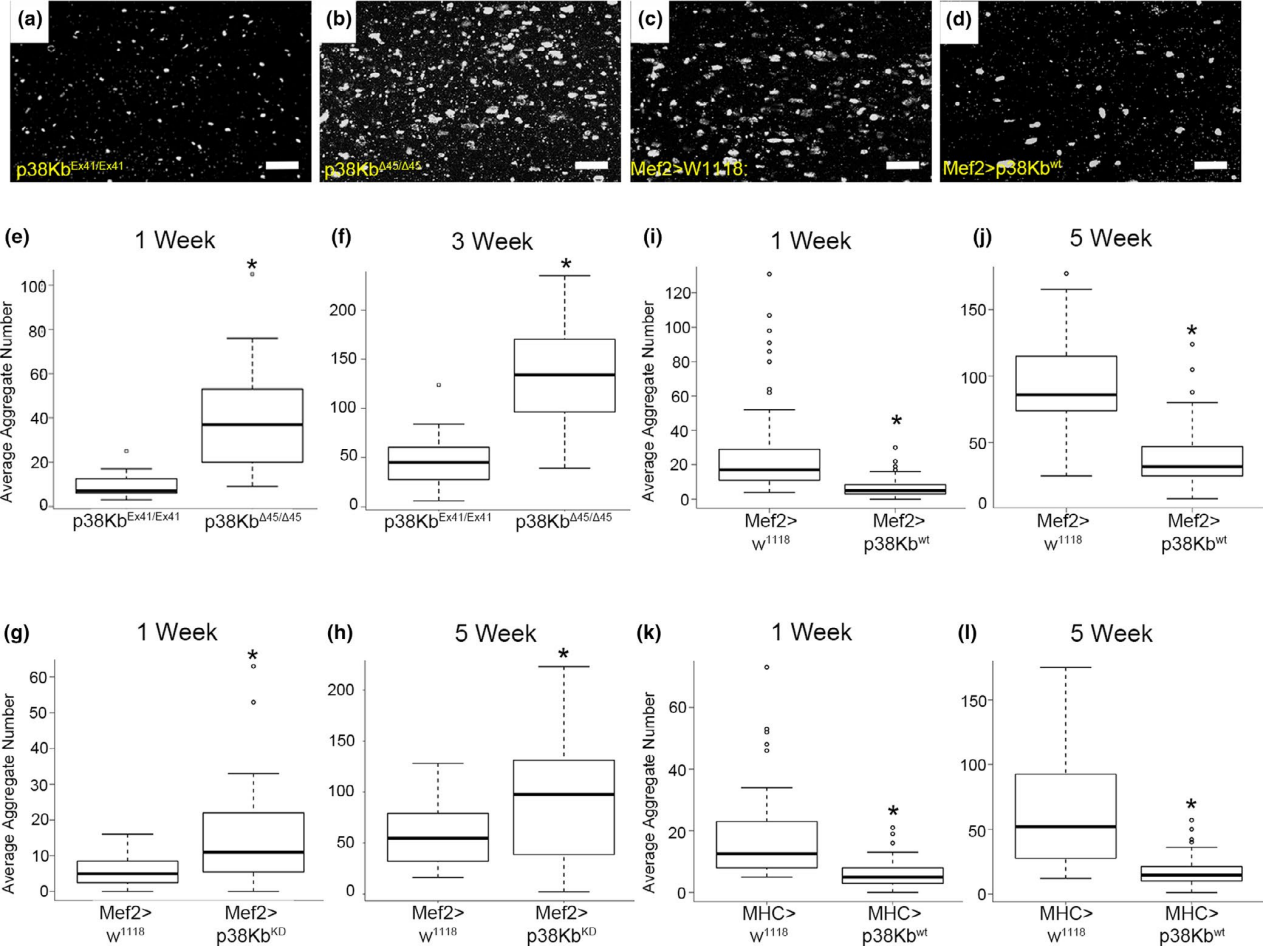
p38Kb regulates age‐dependent protein homeostasis. Confocal micrographs of poly‐ubiquitin‐positive protein aggregates in the adult indirect flight muscle in (a) a precise excision genetic background control p38Kb^Ex41/ Ex41^ and (b) p38Kb ^Δ45/Δ45^ null mutants at 3 weeks of age and in (c) outcrossed Mef2‐GAL4 controls (Mef2>w^11118^) and (d) UAS‐p38Kb^wt^ Mef2‐GAL4 (Mef2>p38Kb^wt^) over‐expression animals at 5 weeks of age. Scale bar equals 6.2 μm. Box–Whisker plots of aggregate number in p38Kb ^Δ45/Δ45^ mutants as compared to p38Kb^Ex41/Ex41^ controls at (e) 1 week and (f) 3 weeks of age. Aggregate number in p38Kb^KD^ Mef2‐GAL4 (Mef2>p38Kb^KD^) and outcrossed Mef2‐GAL4 controls at (g) 1 week and (h) 5 weeks of age. Aggregate number in strong p38Kb over‐expression animals and outcrossed Mef2‐GAL4 controls at (i) 1 week and (j) 5 weeks of age. Aggregate number in moderate p38Kb over‐expression (MHC>p38Kb^wt^) animals and outcrossed MHC‐GAL4 controls (MHC>w^1118^) at (k) 1 week and (l) 5 weeks of age

p38Kb null mutant animals (p38Kb^Δ45/Δ45^, a deletion of the p38Kb gene) exhibit age‐dependent locomotor behavior defects and have a 48% lifespan reduction (Vrailas‐Mortimer et al. 2011). In addition, biochemical analysis suggests that p38Kb null mutants have increased levels of insoluble poly‐ubiquitinated proteins in the thoracic musculature of aged animals as observed by immunoblot analysis (Belozerov et al., 2014). However, protein aggregate size and distribution have not been visualized in the p38Kb mutants nor is it known whether augmenting p38Kb activity in muscles leads to a change in protein homeostasis. Therefore, we analyzed how p38Kb expression influences protein aggregation. We find that p38Kb null mutants have an increased number of protein aggregates in the adult indirect flight muscle at 1 week and 3 weeks of age (Figure 1a,b,e,f, Table S1) and increased aggregate size with age (Figure S1A,B, Table S1) as compared with a genetic background control (p38Kb^Ex41/Ex41^, a precise excision allele). Furthermore, transgenic inhibition of p38Kb in the muscle using a dominant negative kinase dead construct (p38Kb^KD^, Vrailas‐Mortimer et al., 2011) also results in a significant increase in aggregate number; however, aggregate size was not affected (Figure 1g,h, Figure S1C,D, and Table S2). Conversely, as both strong and moderate levels of p38Kb over‐expression lead to an increased lifespan (37% and 14% extension, respectively) (Vrailas‐Mortimer et al., 2011), we tested if p38Kb over‐expression also affects protein aggregation. We find that both strong over‐expression of p38Kb in the adult muscle using the Mef2‐GAL4 driver (Figure 1c,d,i,j, Figure S1E,F, and Table S3) and moderate over‐expression of p38Kb using the MHC‐GAL4 driver (Figure 1k‐l, Figure S1G,H, and Table S4) leads to decreased protein aggregate number and size throughout the lifespan. It has been hypothesized that protein aggregate accumulation may be toxic, potentially explaining the decreased lifespan and age‐dependent locomotor abnormalities in p38Kb^Δ45^ null mutants and the increased longevity in the p38Kb over‐expression animals. Though we find that p38Kb also regulates aggregate size, it is unclear how aggregate size influences protein aggregate toxicity.

### 2.2 p38Kb mediates age‐dependent phenotypes through autophagy

**FIGURE 2:**
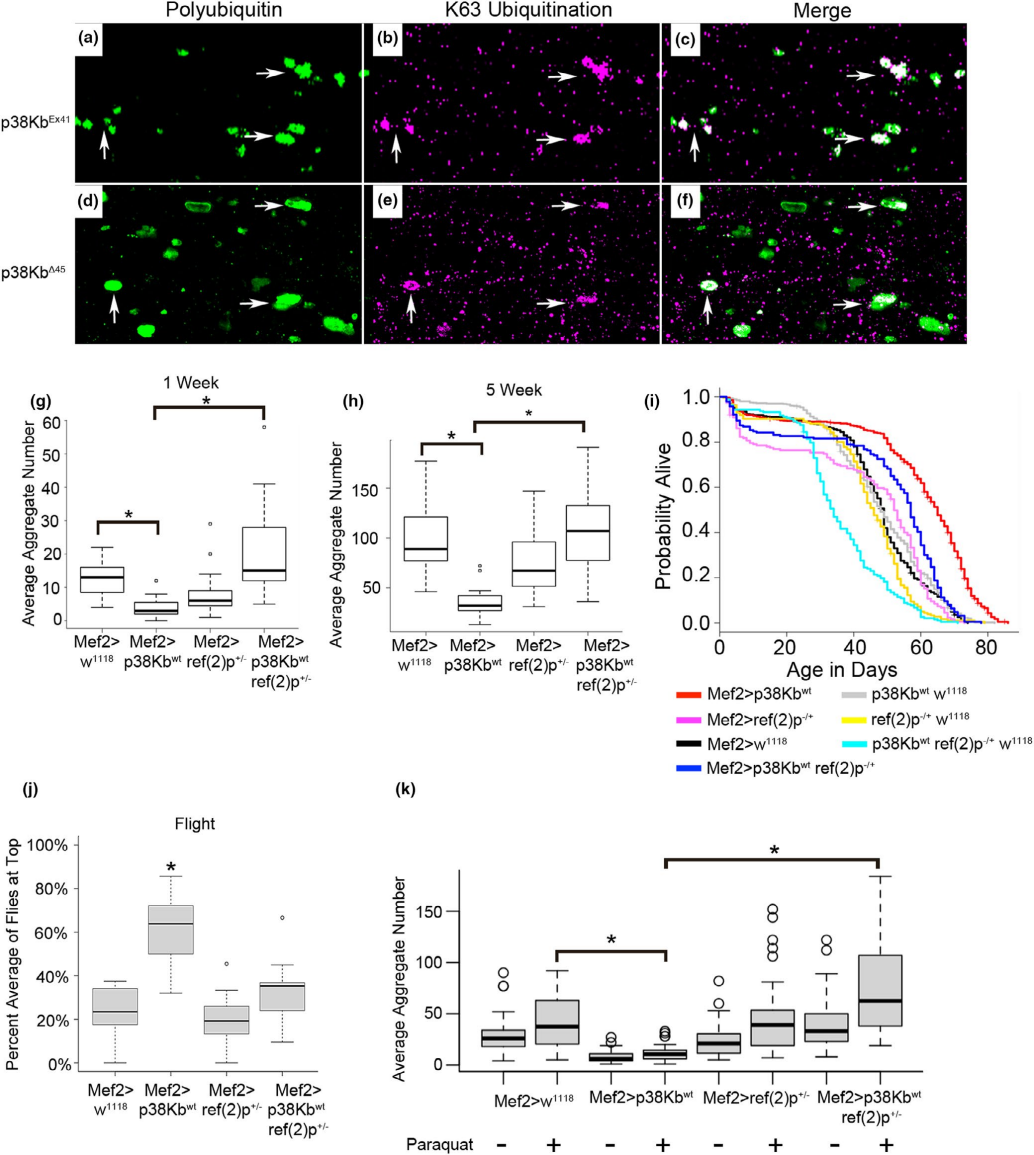
p38Kb regulates aging phenotypes through *ref(2)p*. (a‐c) A subset of poly‐ubiquitinated protein aggregates (green) in 3 week old p38Kb^Ex41/Ex41^ control muscle contain K63 ubiquitinated (magenta) proteins (white arrows). (d‐f) K63 ubiquitin‐positive protein aggregates are also observed in 3‐week‐old p38Kb^Δ45/Δ45^ mutant muscle. Poly‐ubiquitin‐positive protein aggregate number in *ref(2)p* heterozygous mutant backgrounds at (g) 1 week and (h) 5 weeks. Loss of a single copy of *ref(2)p* prevents the p38Kb mediated reduced protein aggregation at 1 week and 5 weeks of age. (i) Over‐expression of p38Kb leads to a lifespan extension (red line) as compared to controls (gray and black lines). Loss of a single copy of *ref(2)p* blocks this lifespan extension (blue line). (j) Flight ability measured by the percentage of flies at the top of the chamber after free fall. Over‐expression of p38Kb leads to improved flight at 1 week of age, which is prevented by loss of a single copy of *ref(2)p*. (k) Number of poly‐ubiquitin‐positive protein aggregates in response to oxidative stress. Paraquat exposure leads to a significant increase in protein aggregation for all genotypes as compared to controls. Over‐expression of p38Kb is protective and requires *ref(2)p* for this protective effect. Asterisks denotes a *p*‐value of ≤0.001 for all panels

In order to determine what mechanism plays a role in the clearance of poly‐ubiquitinated protein aggregates, we first tested what type of ubiquitin linkage is present in the aggregates in wild‐type flies. In particular, K63‐linked ubiquitination has been shown to facilitate the formation of aggregates (Lim et al., 2005; Olzmann et al., 2007; Yung et al., 2016) that are cleared through autophagy (Hao et al., 2013; Tan et al., 2008; Yung et al., 2016). We find that a subset of aggregates from both aged wild‐type and p38Kb mutant flies contain K63‐linked ubiquitinated proteins (Figure 2a‐f), suggesting that at least some of these aggregating muscle proteins are targeted for degradation through autophagy.

Poly‐ubiquitinated protein aggregates can be degraded through selective autophagy in which the adaptor protein p62/ref(2)p promotes the packaging and delivery of poly‐ubiquitinated proteins to the autophagosome (Katsuragi et al., 2015). If p38Kb requires selective autophagy to mediate protein homeostasis, then loss of *ref(2)p* will block the p38Kb mediated decreased aggregation phenotype. Since *ref(2)p* homozygous mutants are viable, this suggests that flies have another compensatory mechanism to regulate protein homeostasis. In order to avoid the effects of a secondary mechanism, we utilized *ref(2)p* heterozygous flies as a sensitized background that would allow us to determine the genetic relationship between p38Kb and *ref(2)p*. We find that loss of a single copy of *ref(2)p*, which allows for analysis of dominant interactions, results in fewer poly‐ubiquitin‐positive protein aggregates (Figure 2g,h and Table S5), which may reflect compensation by other protein clearance mechanisms with aging, especially as homozygous *ref(2)p* mutants are viable. When a single copy of *ref(2)p* is removed in the p38Kb over‐expression background, this prevents the reduced protein aggregation observed in the p38Kb over‐expression animals at both young and old ages (Figure 2g,h and Table S5). As loss of a single copy of *ref(2)p* results in a dominant interaction, this suggests that p38Kb is sensitive to the levels of ref(2)p in order to promote the degradation of protein aggregates and that p38Kb is acting through *ref(2)p* to regulate protein aggregation.

As the level of protein aggregation may influence lifespan (Demontis & Perrimon, 2010; Konstantinidis & Tavernarakis, 2021; Pras & Nollen, 2021), we next tested if p38Kb‐mediated lifespan extension also requires *ref(2)p*. We find that loss of a single copy of *ref(2)p* has no effect on lifespan as compared with the wild‐type GAL4 control (Figure 2i and Table S6). However, loss of *ref(2)p* blocks the lifespan extension of p38Kb over‐expression (Figure 2i and Table S6). As locomotor function decreases with age, we tested if *ref(2)p* is also required for p38Kb mediated locomotor functions. We find that over‐expression of p38Kb results in improved flight behavior, which is prevented by loss of *ref(2)p* (Figure 2j). These data suggest that *ref(2)p* mediated degradation of damaged/misfolded proteins is required for p38Kb mediated reduced protein aggregation, lifespan extension, and improved flight behavior.

As p38Kb is a regulator of oxidative stress, which has also been linked to increased protein aggregation (Korovila et al., 2017; Lévy et al., 2019), we tested if exposure to the oxidizing agent paraquat can induce protein aggregation in the adult flight muscle. We find that control flies fed 20 mM paraquat had increased protein aggregation and that over‐expression of p38Kb protects against this oxidative stress‐induced protein aggregation (Figure 2k and Table S7). We also find that paraquat leads to increased protein aggregation in flies with a loss of a single copy of *ref(2)p*, but this increase is comparable to that of the wild‐type controls (Figure 2k and Table S7). Similar to what we find under normal conditions, loss of *ref(2)p* prevents the protective effect of p38Kb over‐expression during oxidative stress (Figure 2k and Table S7). Therefore, p38Kb mediates protein homeostasis in response to aging and oxidative stress through an interaction with *ref(2)p*.

### 2.3 p38Kb colocalizes and physically interacts with starvin in the adult flight muscle

**FIGURE 3:**
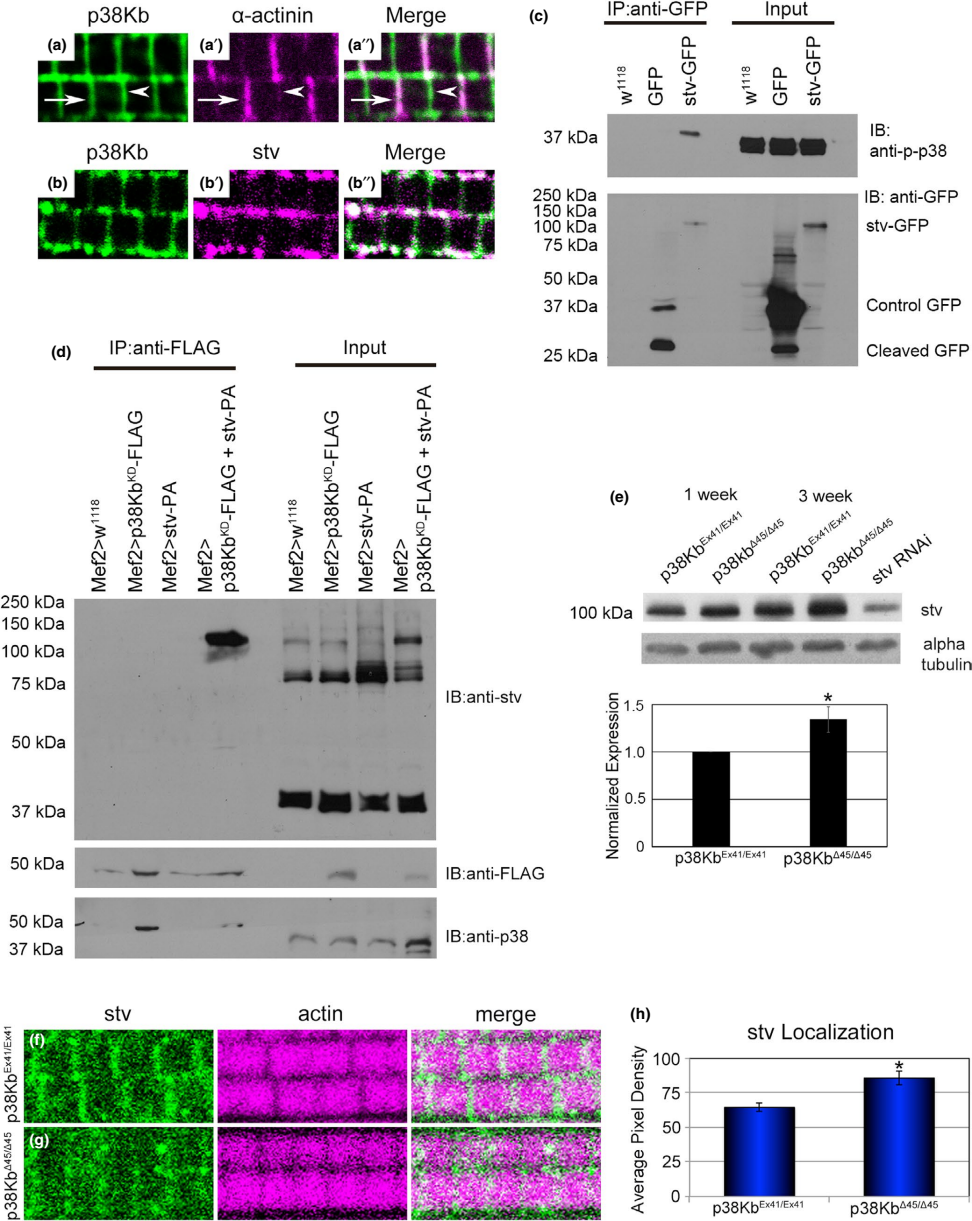
p38Kb and stv colocalize and physically interact (a‐d) Localization of a FLAG‐tagged p38Kb (green in a‐b and a’’‐b’’) in the adult indirect flight muscle. (a) FLAG‐tagged p38Kb localizes to the Z‐disk (arrows) as exhibited by colocalization with the Z‐disk protein alpha‐actinin (magenta, a’ and a’’), as well as the M‐line (arrowheads). (b) p38Kb colocalizes with endogenous stv (magenta, b’‐d’) at the Z‐disk. (c) Muscle lysates expressing control, UAS‐mito‐GFP, or endogenously tagged stv were immunoprecipitated using anti‐GFP coated beads. Endogenous phoshop‐p38K was pulled down by stv. Anti‐GFP was used to confirm to the pull down of both mito‐GFP alone and stv‐GFP. (d) Muscle lysates expressing control and Mef2‐GAL4 UAS‐p38Kb^KD‐FLAG^ in a wildtype background or in a background also expressing UAS‐stv‐PA were immunoprecipitated using anti‐FLAG coated beads. stv was pulled down by p38Kb^KD‐FLAG^. Anti‐FLAG and anti‐total‐p38 were used to confirm pull down of p38Kb^KD‐FLAG^. (e) Immunoblots of stv from 1 and 3 week old muscle lysates of control and p38Kb mutants. Asterisks denotes a *p*‐value of ≤0.05. (f) stv localizes to the adult muscle Z‐disk and M‐line (white arrows) in control animals. (g) stv localization is disrupted in p38Kb mutants. (h) Quantification of average pixel density. Asterisk denotes a *p*‐value of 0.0020348

In order to better understand the role of p38Kb in protein homeostasis, we first determined where in the muscle p38Kb localizes and find that a FLAG‐tagged p38Kb (green) colocalizes with the Z‐disk marker alpha‐actinin and is also present at the M‐line (Figure 3a). The muscle Z‐disk is an area of high protein turnover, and one protein quality control mechanism that localizes to the Z‐disk in mice and adult flies is the Chaperone‐Assisted Selective Autophagy (CASA) complex (known as the BAG‐3 mediated selective autophagy complex in mammals) (Arndt et al., 2010; Chakraborty et al., 2019; Ulbricht et al., 2013). In adult *Drosophila* muscle, the CASA complex has been reported to consist of the Hsp70 homologue Hsc70‐4, the HspB8 homologue CG14207 and the BAG‐3 homologue starvin (stv) (Arndt et al., 2010). stv is a nucleotide exchange factor that binds to both Hsp70 and HspB8 as well as a variety of target proteins that need refolding (Brooks et al., 2020; Carra et al., 2008; Doong et al., 2002; Gamerdinger et al., 2011; Guilbert et al., 2018; Gupta et al., 2019; Ulbricht et al., 2013). As stv has been shown to be required for muscle functions (Arndt et al., 2010; Brooks et al., 2020; Coulson et al., 2005), we tested if p38Kb and stv colocalizes and find that they colocalize at both the Z‐disk and M‐line (Figure 3a,b). We next tested if p38Kb physically interacts with stv in the adult muscle. We utilized an endogenously GFP‐tagged stv in which GFP is spliced in as a new exon of the endogenous gene. This results in a GFP fusion protein that is expressed in the same pattern as endogenous stv (Arndt et al., 2010 and Figure 3b’). stv produces seven isoforms resulting in five unique proteins (FlyBase), and the stv‐GFP captures the three longest isoforms (stv‐PA, PE, and PF, encoded by the alternatively spliced stv‐RA, RE, and RF transcripts). We immunoprecipitated either a control GFP (GFP tagged with a mitochondrial localization signal expressed in the muscle using Mef2‐GAL) or stv‐GFP from muscle lysates and find that stv pulls down with endogenous phosphorylated p38K, which is the active form of p38K (Figure 3c). We then performed reverse immunoprecipitation experiments, in which we utilized a FLAG‐tagged p38Kb kinase dead construct (UAS‐p38Kb^KD^ Mef2‐GAL4). p38Kb^KD^ is able to be activated and bind to a target but cannot phosphorylate it, leading to a delayed release of the target (Hattori et al., 2013) and increasing the possibility of capturing transient interactions. We expressed p38Kb^KD^ in a wild‐type background or in a background that also over‐expresses the stv‐PA isoform. stv‐PA is predicted to be ~66 kDa; however, we find that it is about 75 kDa with a minor band at ~100 kDa (input lanes, Figure 3d), suggesting that it is post‐translationally modified. Furthermore, co‐over‐expression with p38Kb^KD^ results in a shift from the 75 kDa form of stv to the ~100 kDa form (input lanes, Figure 3d). In addition, we find that p38Kb^KD^ co‐immunoprecipitates with stv‐PA (Figure 3d). These data suggest that the kinase activity of p38Kb is important for processing of post‐translationally modified stv‐PA.

### 2.4 p38Kb is required for proper localization of stv in the muscle

As we find that p38Kb interacts with stv, we next tested if p38Kb regulates stv protein levels and/or localization. We find that in p38Kb null mutants, endogenous stv protein levels are increased (Figure 3e), and stv is more diffusely located throughout the muscle but is still able to localize to the Z‐disk and M‐line in (Figure 3f‐h), suggesting that its localization is partially impaired in the absence of p38Kb. Furthermore, we find that the localization of HspB8/CG14207, which co‐immunoprecipitates with stv in Drosophila cell culture and colocalizes with stv at the Z‐disk in the adult muscle (Arndt et al., 2010) is unaffected by loss of p38Kb (Figure S2). This suggests that loss of p38Kb specifically affects stv protein levels and localization.

### 2.5 p38Kb acts with stv to regulate protein homeostasis

**FIGURE 4:**
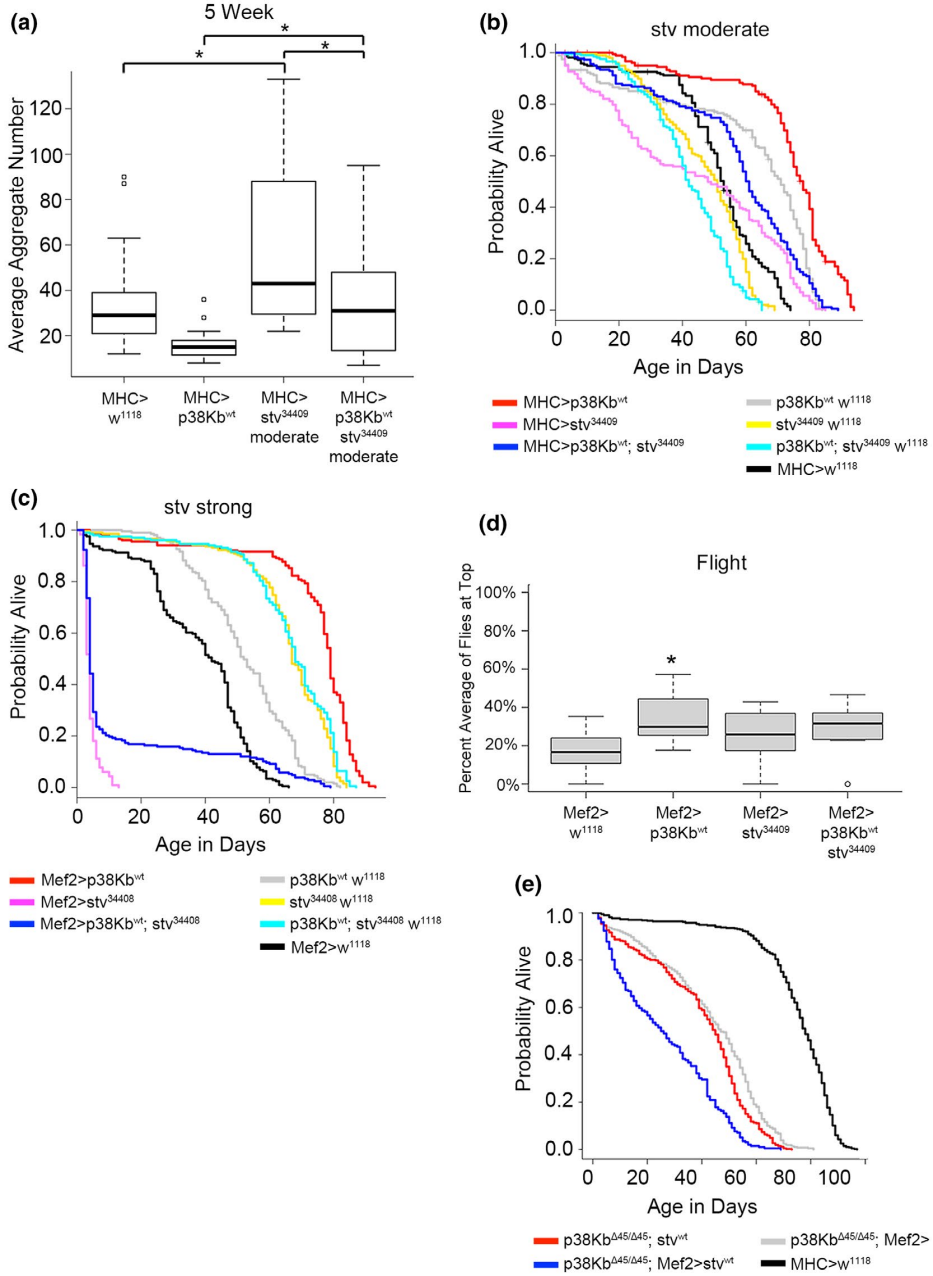
p38Kb genetically interacts with stv to regulate protein homeostasis and lifespan. (a) Protein aggregate number in the moderate stv knockdown background using MHC‐GAL4 at 5 weeks. Asterisks denote a *p*‐value of ≤0.001. (b) Moderate over‐expression of p38Kb (red line) results in an increased lifespan as compared to the MHC‐GAL4 controls and p38Kb transgene control (black line and gray lines, respectively). Moderate knockdown of stv using the MHC‐GAL4 results in a decreased lifespan (pink line compared to yellow and black lines) and prevents p38Kb over‐expression lifespan extension (compare pink line to blue line). (c) Strong over‐expression of p38Kb (red line) results in an increased lifespan as compared to the Mef2‐GAL4 controls and p38Kb transgene control (black line and gray lines, respectively). Strong knockdown of stv using the Mef2‐GAL4 results in a decreased lifespan (pink line compared to yellow and black lines) and prevents p38Kb over‐expression lifespan extension (compare pink line to blue line). (d) Flight behavior was analyzed using the escape from free fall assay. Percent average of flies at the top of the chamber were measured. Asterisk denotes a *p*‐value of 0.0469 (e) Over‐expression of stv in the p38Kb mutant background results in a further reduction of lifespan as compared to p38Kb mutant controls (compare blue line to red and gray lines)

As BAG‐3/stv provides both the specificity to the CASA complex and is involved in the handoff of damaged proteins to p62/ref(2)p for autosomal degradation (Behl, 2016; Gamerdinger et al., 2009, 2011; Zhang & Qian, 2011), we tested for genetic interactions between p38Kb and *stv*. *stv* null mutants have impaired locomotor functions, muscle degeneration, and early lethality (Arndt et al., 2010; Coulson et al., 2005). Due to the severity of these null phenotypes, we utilized *stv* RNAi lines to generate an allelic series of *stv* loss of function in the muscle. We find that weak inhibition of stv (UAS‐stv RNAi^34408^ MHC‐GAL4) had no effect on protein aggregation (Table S8). However, moderate inhibition of stv (UAS‐stv RNAi^34409^ MHC‐GAL4) results in increased protein aggregate number and size (Figure 4a, Figure S3 and Table S9) and results in a decrease in lifespan particularly in the first half of life as compared to outcrossed controls (Figure 4b and Table S10). Strong inhibition of *stv* (UAS‐stv RNAi^34408^ Mef2‐GAL4) leads to a severely reduced lifespan of ~4 days on average (Figure 4c and Table S11).

We find that inhibition of *stv* blocks both the reduced protein aggregate number and size mediated by p38Kb over‐expression (Figure 4a and Figure S3 and Table S9) and also prevents the p38Kb mediated lifespan extension (Figure 4b,c and Tables S10 and S11). We next tested if p38Kb and *stv* genetically interact to regulate age‐dependent locomotor functions and find that moderate inhibition of *stv* has no effect on flight ability at 5 weeks of age; however, this inhibition is sufficient to prevent p38Kb mediated improved flight ability (Figure 4d). These data demonstrate the p38Kb and *stv* interact to regulate different aspects of aging, including protein homeostasis, lifespan, and locomotor function.

To further explore how p38Kb and stv are interacting with each other, we next tested if over‐expression of stv in p38Kb mutants affects lifespan. We find that over‐expression of stv in the p38Kb mutants leads to a further reduction in the p38Kb mutant lifespan (Figure 4e and Table S13). In addition, over‐expression of stv in the p38Kb mutant background also leads to reduced adult viability (Table S13). This is particularly striking as over‐expression of stv in a wild‐type background does not significantly affect lifespan or viability (Figure S4E and Table S13) as compared to the outcrossed transgene control. Furthermore, this interaction is specific to stv as over‐expression of Hsc70‐4 in the muscle does not affect p38Kb mutant shortened lifespan (Figure S5A, Table S14), even though Hsc70‐4 over‐expression extends lifespan in a wild‐type background (Figure S5B, Table S15). These data suggest that p38Kb may be a limiting factor in regulating the downstream activity of stv.

### 2.6 p38Kb regulates the activity of stv in age‐dependent protein homeostasis

If p38Kb is a limiting factor for stv function, then the combined over‐expression of p38Kb and stv may result in a further beneficial effect. We find that over‐expression of stv alone results in fewer aggregates at young and old ages (Figure S4A,B and Table S16) and smaller aggregates with age (Figure 4d and Table S16) as compared to outcrossed controls. However, co‐over‐expression of p38Kb and stv does not result in a further reduction in aggregate number as compared to over‐expression of p38Kb or stv alone (Figure S4A and Table S16). By 5 weeks of age, co‐over‐expression p38Kb and stv flies have a comparable aggregate number to controls (Figure S4B and Table S16), suggesting that p38Kb and stv co‐over‐expression has more benefits at younger ages on regulating aggregate number. Conversely, p38Kb and stv co‐over‐expression results in a reduction in aggregate size at a young age compared to over‐expression of stv alone (Figure S4C and Table S16), suggesting that p38Kb is a limiting factor for stv function in regulating aggregate size. Unlike with aggregate number, the aggregates remain significantly smaller in size as the flies age in the combined over‐expression background (Figure S4D and Table S16). We also find that co‐over‐expression of p38Kb and stv leads to an additional 5% increase in lifespan relative to p38Kb over‐expression alone (Figure S4E and Table S13). Interestingly, the co‐over‐expression animals show a very similar lifespan to the p38Kb over‐expression alone animals until ~day 50, when the p38Kb alone animals begin to die at a faster rate (Figure S4E). These data suggest that co‐over‐expression of p38Kb and stv provides beneficial effects in early adulthood that continue throughout adulthood leading to increased lifespan despite the presence of wild‐type numbers of protein aggregates. Another possibility is that aggregate size and/or which proteins are aggregating may play a more important role in determining lifespan as compared to overall aggregate number.

As p38Kb over‐expression is also protective against oxidative stress induced protein aggregation, we tested if p38Kb and stv interact to regulate protein homeostasis in response to paraquat exposure. We find that over‐expression of stv is protective against oxidative stress as compared to controls (Figure S5F and Table S17, Dunnett's test *p *= 0.0463). However, we find that over‐expression of stv prevents the protective effect of p38Kb (Figure S5F and Table S17, Dunnett's test *p *< 1e‐04). These data indicate that stv does not interact with p38Kb to regulate protein homeostasis in response to oxidative stress and that p38Kb may be acting through a different ref(2)p mediated autophagy mechanism in response to paraquat.

### 2.7 Lamin protein accumulates in stv RNAi and p38Kb mutants

In order to determine if p38Kb might be playing a role in the hand off of misfolded proteins from stv to ref(2)p, we first needed to identify a protein target of both stv and p38Kb. Since there may be a number of target proteins, we decided to focus on candidate proteins that have links to both BAG‐3/stv and p38K. BAG‐3 in humans accumulates in cytoplasmic inclusions that varied in size in the muscle tissue of Limb‐Girdle Muscular Dystrophy (LGMD) Type 1D patients (Sato et al., 2013). In addition, p38K signaling has been implicated in LGMD (Capanni et al., 2003; Fanzani et al., 2007; Suzuki et al., 2012) with manipulation of p38K improving muscle function in a mouse LGMD model (Suzuki et al., 2012). *Drosophila* have 19 orthologues of LGMD proteins, including homologues of the nuclear envelope protein Lamin A/C (Lamin). Lamin is of particular interest since mutations in Lamin also result in the accelerated aging disorder Hutchinson‐Gilford progeria (De Sandre‐Giovannoli et al., 2003; Eriksson et al., 2003; Mounkes et al., 2003), and it has been shown to aggregate under oxidative stress conditions (Singla et al., 2013). Furthermore, BAG‐3 can target nuclear Lamin B, a paralogue of Lamin A/C, for degradation (Gupta et al., 2019). Mutations in Lamin are also sufficient to induce Lamin protein aggregation and abnormal nuclear morphology in human cell culture, *C*. *elegans*, and *Drosophila* systems (Ahmed et al., 2018; Bank et al., 2011; Barascu et al., 2012; Casasola et al., 2016; Chandran et al., 2019; Dialynas et al., 2015; Eriksson et al., 2003; Hübner, et al., 2006; Hübner et al., 2006; Zaremba‐Czogalla et al., 2012). In *Drosophila*, inhibition of Lamin homologues results in similar phenotypes to p38Kb and/or stv mutants, such as reduced locomotor function and increased activity of the Nrf‐2/Keap‐1 pathway (Chandran et al., 2019; Dialynas et al., 2010, 2015; Li et al., 2016; Muñoz‐Alarcón et al., 2007, Vrailas‐Mortimer et al., 2011). Based on these phenotypic similarities, we tested whether Lamin may be a target for p38Kb and stv in *Drosophila*. Flies have two Lamin homologues, Lamin Dm_0_ and LamC, and we focused on Dm_0_ as it has properties of both mammalian Lamin A/C and Lamin B (Lenz‐Böhme et al., 1997; Muñoz‐Alarcón et al., 2007; Oyston et al., 2018).

In mammals, Lamin proteins are highly post‐translationally modified, resulting in changes in solubility (Brandt et al., 2008; Polychronidou et al., 2010; Rzepecki & Fisher, 2002; Schneider et al., 1999; Smith et al., 1987; Zaremba‐Czogalla et al., 2011, 2012), therefore, we began by characterizing the Lamin Dm_0_ protein in flies. Using a total Lamin Dm_0_ antibody, we find that Lamin Dm_0_ is expressed predominantly as a ~75 kDa protein (Figure S6A‐C). Lamins are phosphorylated by a variety of kinases that can change the solubility of Lamin proteins (Cao et al., 2007; Zaremba‐Czogalla et al., 2012). Using an antibody specific for phospho‐Ser 45 Lamin (Ser 22 in humans), we find that Lamin is phosphorylated in *Drosophila* (Figure S6B) and that the phosphorylated form of Lamin runs at lower molecular weight than the main form of Lam Dm_0_ (Figure S6A), suggesting that Lamins in adult *Drosophila* muscle are further processed. As this phosphorylation site is near the epitope recognized by the total lamin antibody, it may be that lamin phosphorylation obscures the ability of this antibody to detect this form of Lamin.

In addition to phosphorylation, Lamins can also be farnesylated, which is required for Lamin localization to the inner nuclear membrane (Holtz et al., 1989; Kitten & Nigg, 1991; Meshorer & Gruenbaum, 2008; Polychronidou et al., 2010), at the C‐terminal CaaX box. As we observe a minor 100 kDa form of Lam Dm_0_ (Figure S6A,C), we tested if this might be a farnesylated form of Lam Dm_0_. We utilized the Lam^A25^ mutant which has a frameshift that results in the loss of the C‐terminal CaaX box (Patterson et al., 2004). We find that this high‐molecular weight form of Lam Dm_0_ is lost in the Lam^A25^ mutant whereas the predominant 75 kDa band of Lam Dm_0_ is still present (Figure S6A). These data suggest that the high‐molecular weight Lam Dm_0_ we observe is the farnesylated form.

**FIGURE 5:**
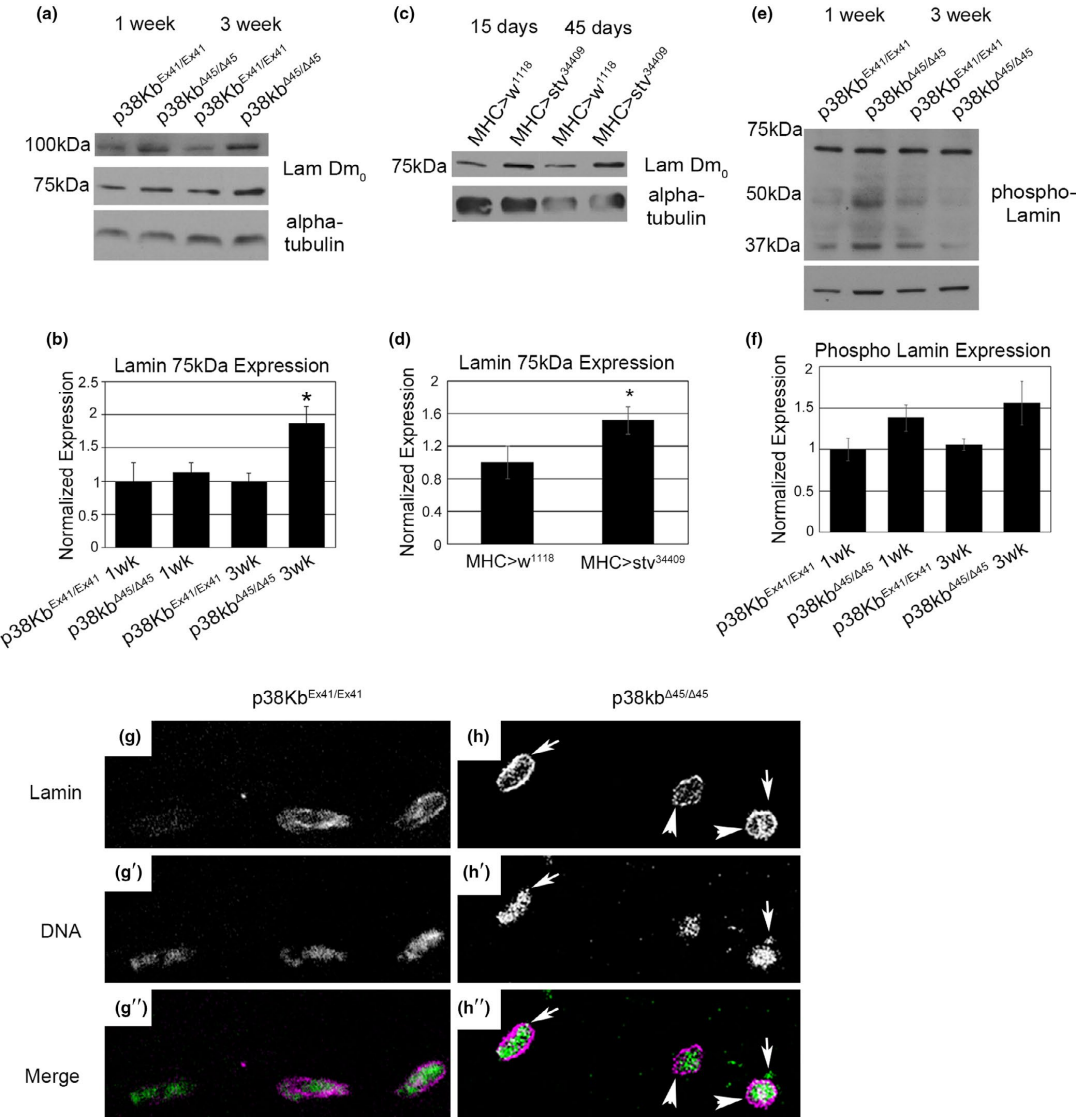
p38Kb and stv regulate Lamin aggregation. (a) Immunoblot analysis of p38Kb^Ex41/Ex41^ control and p38Kb ^Δ45/Δ45^ mutant muscle lysates probed with anti‐Lamin and (b) quantification of the 75 kDa form using densitometry, asterisk denotes *p*‐value = 0.006643. (c) Immunoblot analysis of stv‐RNAi and GAL4 controls flies muscle lysates probed with anti‐Lamin and (d) quantified using densitometry, asterisk denotes p‐value = 0.028. (e) Immunoblot analysis of p38Kb^Ex41/Ex41^ control and p38Kb ^Δ45/Δ45^ mutant muscle lysates probed with anti‐phospho‐Lamin and (f) quantified using densitometry, *p*‐value = 0.09. (g,h) Confocal micrographs of adult muscle from 3 week‐old (g) wild‐type and (h) p38Kb mutants stained for lamin (magenta) and the DNA marker syto24 (green). (g) Nuclei in wild‐type muscle have an elliptical shape, while (h) in p38Kb mutant muscle appear more circular (arrowheads) and there is nuclear leakage (arrows). In addition, the lamin staining is more punctate

If Lamin is a target of p38Kb and stv, then decreased activity of either p38Kb or stv should result in an accumulation of Lamin protein. We first tested if the levels of the main 75 kDa form of Lam Dm_0_ are altered in p38Kb mutants. We find that the levels of Lam Dm_0_, do not change with age in wild‐type controls (Figure 5a,b). However, loss of p38Kb leads to an age‐dependent increase in the predominant form of Lam Dm_0_ protein as compared to age matched controls (Figure 5a,b). Furthermore, we find that inhibition of stv also results in a significant increase in the total amount of the main form of Lam Dm_0_ protein regardless of age (Figure 5c,d). We next wanted to test if post‐translationally modified forms of Lamin are impacted by p38Kb. We find that phospho‐Lamin levels are not significantly altered in the p38Kb mutants (Figure 5e,f), suggesting that p38Kb is not required for Ser45 phosphorylation. We additionally find that loss of p38Kb has no effect on the levels of the farnesylated Lam Dm_0_ (Figure 5a and Figure S6C‐D).

As the p38Kb mutants have increased levels of Lam Dm_0_, one possibility is that this leads to excess Lam Dm_0_ activity that contributes to p38Kb mutant phenotypes. Therefore, we hypothesized that inhibition of Lam Dm_0_ will rescue some of the p38Kb mutant phenotypes. However, whereas knock down of Lam Dm_0_ in wild‐type muscle was viable, we find that the knock down of Lam Dm_0_ in the muscle of p38Kb mutants, using two different RNAi lines and both the Mef2‐ and MHC‐GAL4 muscle drivers, results in organismal lethality. This indicates that p38Kb mutant phenotypes are not driven by an excess of Lam Dm_0_ activity but rather that this increased Lam Dm_0_ protein is either nonfunctional or acts as a dominant negative. Thus, knock down of Lam Dm_0_ in the p38Kb mutants leads to a further reduction in functional Lam Dm_0_ protein and subsequent lethality. Therefore, we tested if Lam Dm_0_ localization in the adult flight muscle is also disrupted by loss of p38Kb. We find that in p38Kb mutants Lam Dm_0_ staining is more punctate (Figure 5h), suggesting that it is no longer forming a supportive meshwork for the nuclear envelope. Furthermore, we find that there is DNA leakage from the nuclei in the p38Kb mutants (Figure 5h’), similar to what has been observed in LamC and Lam Dm_0_ mutants (Schulze et al., 2005, 2009; Uchino et al., 2013). In addition, we also find that the nuclei are more rounded as compared to age matched controls (Figure 5g,h). Therefore, in the absence of p38Kb, both Lam Dm_0_ localization and nuclear integrity are disrupted, indicating a functional relationship between p38Kb and Lam Dm_0_.

### 2.8 Lamin protein aggregates in p38Kb mutants

To investigate if Lam Dm_0_ is accumulating in the protein aggregates, we performed fractionation experiments in which we separated cytosolic proteins using sucrose density centrifugation. We isolated 10 fractions in addition to the insoluble pellet. We find that the pellet contains both poly‐ubiquitinated proteins and K63 conjugated ubiquitinated proteins (Figure S7A,B), which is characteristic of the protein aggregates that we identified in whole mount muscle tissue (Figures 1 and 2). In addition, the nuclear pore complex protein Megator was not found in any of the fractions or the pellet, demonstrating that there were no contaminating membranes or nuclei in the preparations (Figure S7C).

**FIGURE 6:**
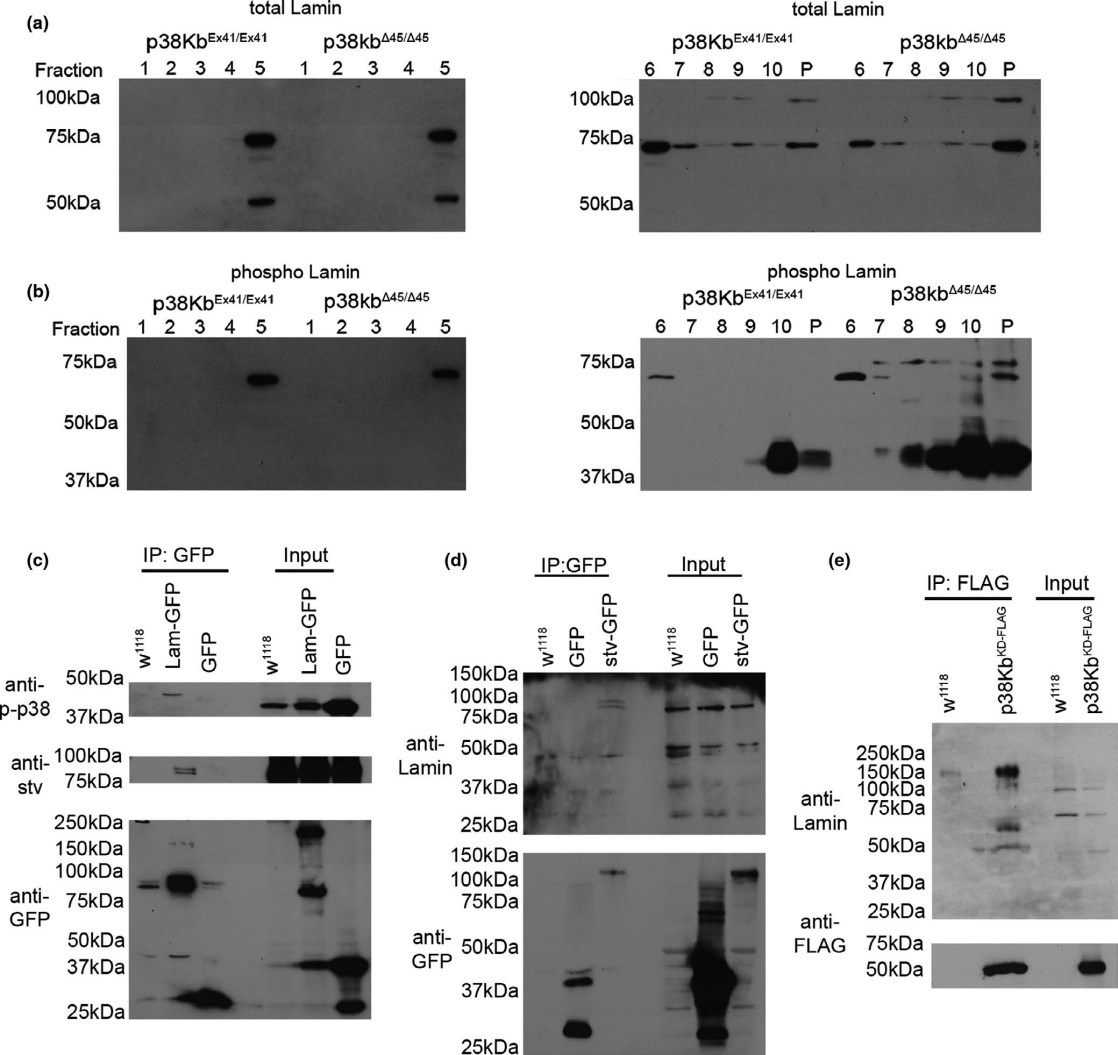
Lamin aggregates in p38Kb mutants and co‐immunoprecipates with p38Kb and stv. (a,b) Immunoblots of sucrose gradient fractions from 3 week old p38Kb^Ex41/Ex41^ control and p38Kb ^Δ45/Δ45^ mutant muscle. (a) The main species of Lamin (~75 kDa) is mainly found in fractions 5–7 with some in the pellet, and the farnesylated form of Lamin (~100 kDa) is mostly found in the pellet in controls. In the p38Kb mutants, there is a further accumulation of Lamin and farnesylated Lamin in the pellet. (b) Phosphorylated Lamin is also found predominantly in fractions 5–7 in controls and accumulates in the pellet in p38Kb mutants. (c‐d) Muscle lysates with control, over‐expression of mito‐GFP, and (c) over‐expression of Lam Dm_0_‐GFP or (d) endogenously tagged stv were immunoprecipitated using anti‐GFP coated beads. (c) Endogenous phoshop‐p38K and stv were pulled down by Lam Dm_0_. (d) Lam Dm_0_ pulled down with stv. Anti‐GFP was used to confirm to the pull down of both mito‐GFP alone, Lam‐GFP, and stv‐GFP. (e) Muscle lysates expressing control and Mef2‐GAL4 UAS‐p38Kb^KD‐FLAG^ in a wildtype background were immunoprecipitated using anti‐FLAG coated beads. Endogenous Lam Dm_0_ was pulled down by p38Kb^KD‐FLAG^. Anti‐FLAG was used to confirm pull down of p38Kb^KD‐FLAG^

We find that in controls, the 75 kDa form of Lamin Dm_0_ is predominantly found in fractions 5–7 and also in the aggregate containing pellet (Figure 6a), Furthermore, we find that the farnesylated form of Lam Dm_0_ is predominantly found in the pellet (Figure 6a). Interestingly, we observe a low molecular weight from of Lam Dm_0_ mainly restricted to fraction 5 (Figure 6a). We next tested how loss of p38Kb affects the aggregation of Lam Dm_0_ and find that p38Kb mutants have decreased Lam Dm_0_ in fractions 5–7 with a concurrent increase of both the main and farnesylated species of Lam Dm_0_ in the pellet (Figure 6a). This suggests that Lam Dm_0_ is a target of p38Kb and that loss of p38Kb results in increased aggregation of Lam Dm_0_. As we do not observe a consistent effect on levels of farnesylated Lam Dm_0_ due to either stv inhibition or loss of p38Kb, this suggests that p38Kb mediates farnesylated Lam Dm_0_ aggregation rather than its overall levels.

We next examined if phosphorylated Lamin also aggregates. We find that in controls, phosphorylated Lamin is present in factions 5–7 with low amounts in the pellet (Figure 6b). We also observe a low molecular weight phospho‐Lamin form that appears in the pellet (Figure 6b). In the p38Kb mutants, phospho‐Lamin expression is reduced in fractions 6 and 7 and accumulates in the pellet (Figure 6b). Furthermore, increasingly smaller low molecular weight species of phospho‐Lamin are present in the p38Kb^Δ45^ mutants including the pellet (Figure 6b), suggesting that loss of p38Kb prevents the effective clearance of these Lamin cleavage forms. Interestingly, loss of the Lamin CaaX box does not affect the formation of these lower molecular weight forms; however, it does result in an increase in the phosphorylation of full‐length Lamin (Figure S6A). Additionally, we do not detect phosphorylation‐positive high‐molecular weight forms of Lamin (Figure 6b), suggesting that farnesylated Lamin is not phosphorylated at Ser45. Overall, these data suggest that Lamin is processed in a variety of different ways in the adult muscle and these different forms are prone to aggregation in the absence of p38Kb.

### 2.9 Lamin physically interacts with stv and p38Kb

To determine if Lam Dm_0_ is a direct target of stv and p38Kb, we over‐expressed GFP‐tagged Lam Dm_0_ in the muscle and were able to pull‐down endogenous stv and phosphorylated p38K (Figure 6c). We also immunoprecipitated endogenously GFP‐tagged stv from adult *Drosophila* muscle and probed for endogenous Lam Dm_0_. We find that both the predominant and farnesylated forms of Lam Dm_0_ co‐immunoprecipitate with stv (Figure 6d). Furthermore, we expressed p38Kb^KD^ in the muscle and found that p38Kb co‐immunoprecipitates with several forms of Lam Dm_0_ including two smaller forms of ~60 and 50 kDa. Interestingly, we do not observe p38Kb interacting with the predominant 75 kDa form but rather with a series of higher molecular weight forms (Figure 6e). As expression of p38Kb^KD^ in the muscle is sufficient to increase protein aggregation (Figure 1), these high‐molecular weight forms of Lam Dm_0_ may correspond to poly‐ubiquitinated forms that are being targeted for degradation.

### 2.10 Model of p38Kb mediated protein homeostasis

We have developed a model in which misfolded proteins such as Lam Dm_0_ are targeted by stv for degradation through autophagy. In this model, those proteins that cannot be refolded are tagged with poly‐ubiquitin to signal their degradation through the autophagosome/lysosome pathway. These poly‐ubiquitinated proteins are handed off by stv to ref(2)p in a process mediated by activated p38Kb. We hypothesize that this hand off is a rapid process as we were unable to detect the higher molecular weight poly‐ubiquitinated Lamin binding to stv but were only able to capture these transient interactions using the p38Kb^KD^ construct. This model explains our finding that in p38Kb mutants these poly‐ubiquitinated proteins accumulate and then form aggregates, and how over‐expression of p38Kb would lead to increased efficiency of the poly‐ubiquitinated proteins being targeted to the autophagosome. In agreement with our model, full‐length Lamin Dm_0_ would bind to stv for refolding and release, or for poly‐ubiquitination and transfer to ref(2)p for degradation through an interaction with p38Kb.

## 3 DISCUSSION

We find that the aging gene p38Kb regulates age‐dependent protein homeostasis through an interaction with stv/BAG‐3 and is acting at a step between the poly‐ubiquitination of an un‐foldable target and its transfer to ref(2)p for autolysosomal mediated degradation. Flies only have one BAG domain containing protein, while in humans there are six (BAG1‐6). All six human BAG proteins can bind to Hsp70 through the BAG domain, and their other protein domains are used to bind to specific protein targets or other chaperones to promote distinct cellular functions (Doong et al., 2002). In *Drosophila*, the *stv* gene produces seven transcripts and five unique protein isoforms (FlyBase), though the functions of these stv isoforms are unknown. We have found that p38Kb co‐immunoprecipitates with the stv‐PA protein isoform and possibly the stv‐PE and PF isoforms as well, suggesting that at least one of these isoforms of stv is involved in regulating age‐dependent protein homeostasis, and targets Lam Dm_0_ for degradation in the adult flight muscle. Furthermore, different stv isoforms may interact with specific chaperones such as HspB8/CG14207 and HspB8/Hsp67Bc to form different CASA complex subtypes in specific tissues/cell types or under different conditions. For example, stv can co‐immunoprecipitate with HspB8/Hsp67Bc in *Drosophila* S2 cell culture and eye tissue, which consists mostly of photoreceptor neurons (Carra et al., 2010). Several studies on stv have focused on embryonic and larval muscle (Brooks et al., 2020; Coulson et al., 2005) and stv colocalizes with HspB8/Hsp67Bc in the larval muscle (Carra et al., 2010). However, in the adult flight muscle stv has only been characterized by an interaction with a CASA complex including HspB8/CG14207 (Arndt et al., 2010). We therefore predict that p38Kb and stv are acting through the HspB8/CG14207 containing CASA complex in the adult flight muscle.

We find that p38Kb is important for the proper localization of stv to the Z‐disk but does not affect the localization of the stv binding partner and adult flight muscle CASA complex member HspB8/CG14207. This suggests that p38Kb may play a role in maintaining the interaction between stv and the CASA complex which may be necessary for target transfer to ref(2)p. stv has a conserved MAPK docking site as well as eight potential p38K phosphorylation sites. Furthermore, the mammalian ref(2)p homolog p62 has been shown *in vitro* to bind to mammalian p38K through two domains (Saito et al., 2008), which are partially conserved in flies. Therefore, one possibility is that p38Kb mediated phosphorylation of stv facilitates the localization of stv to form a functional CASA complex at the Z‐disk, where damaged proteins are rapidly turned over. Another possibility is that p38Kb binds to stv and ref(2)p, and that the phosphorylation of stv is required for target hand‐over to ref(2)p so that in the absence of p38Kb, stv cannot transfer targets to ref(2)p. A consequence of this may be that stv and the protein target together are released from the CASA complex, leading to mislocalization of stv. As we do not detect a decrease in phospho‐Lamin in the p38Kb mutants or find that p38Kb can pull‐down phospho‐Lamin, it is unlikely that p38Kb is directly phosphorylating the target proteins as a part of the stv‐ref(2)p hand‐over process. However, as more about stv function in the adult flight muscle is uncovered, we may find that p38Kb and stv are working through an alternative protein quality control mechanism.

We find that the protein aggregates in the adult fly muscle contains K63‐ubiquitinated proteins and were unable to detect K48 ubiquitinated proteins in the aggregates (data not shown). However, there is not complete overlap between K63‐ubiquitination and the poly‐ubiquitin staining used to mark the aggregates. One possibility is that all the proteins in the aggregates have K63‐linked ubiquitination, but the epitope recognized by the antibody is buried within the aggregate. Another possibility is that the aggregates contain proteins with different types of ubiquitin linkages, and the antibody used was unable to detect the K48 linkage of aggregating proteins. As little is known about which proteins are aggregating or which ubiquitin modifications they have, further analysis of the aggregates will be necessary to understand how these aggregates form.

How protein aggregation contributes to aging and disease has been an area of great interest. One outstanding question is if protein aggregation is a consequence or cause of aging. It has been hypothesized that protein aggregates accumulate with age as the amount of damaged or misfolded proteins increase. However, it is not clear whether or not these aggregating proteins are toxic leading to tissue dysfunction and a disease state. Previous studies have found that long‐lived fly strains such as over‐expression of Foxo or parkin result in reduced protein aggregate formation (Demontis & Perrimon, 2010; Rana et al., 2013). Therefore, we hypothesized that decreased protein aggregation would lead to a lifespan extension, while increased protein aggregation would lead to a reduced lifespan. As expected, we find that the short‐lived p38Kb mutants, which exhibit premature locomotor behavior defects (Vrailas‐Mortimer et al., 2011), have large and numerous protein aggregates. We also find that over‐expression of p38Kb leads to decreased protein aggregation and increased lifespan and improved flight.

However, while inhibition of stv results in increased aggregation and decreased lifespan, over‐expression of stv leads to decreased aggregate number without a concurrent lifespan extension. These data suggest that protein aggregation and lifespan may be separable processes. In addition, we find that stv does not interact with p38Kb to reduce paraquat induce protein aggregation, suggesting that stv may have specific functions within maintaining cellular and organismal health.

We also find that co‐over‐expression of p38Kb and stv does not lead to decreased protein aggregation at older ages whereas over‐expression of either p38Kb or stv alone does. One possibility is that there is an age‐dependent decrease in another key protein that plays a role in overall protein turnover. With aging, p38Kb and stv may be targeting specific proteins for degradation, but the amount of this third protein is not sufficient to handle the influx of targeted proteins, leading to the accumulation of aggregates in older ages. However, as p38Kb and stv are still promoting targets for degradation, specific target proteins are being cleared leading to smaller aggregates and maintaining organismal health.

Another potential interpretation of these results is that aggregate number is not as critical for lifespan extension, but rather which protein species are aggregating or the number of aggregating proteins, particularly early in life, is critical for lifespan extension. Therefore, the clearance of specific protein species that are toxic to the cell or limiting the accumulation of these protein species may improve organismal health. We find that stv over‐expression only results in decreased aggregate size in older animals; however, co‐over‐expression of p38Kb and stv leads to reduced aggregate size at both young and old ages and leads to a further increase in lifespan. Thus, the turnover of a specific toxic protein or subset of proteins early in life may lead to a reduction in the exposure to these toxic species and a lifespan extension. Furthermore, it has been hypothesized that protein aggregates may be protective in some instances. For example in Alzheimer's disease it has been hypothesized that the soluble form of Amyloid‐β is toxic and that the formation of aggregates protects against this toxicity (Hayden & Teplow, 2013; Horváth et al., 2019; Kayed et al., 2003; Lesné et al., 2006; Walsh et al., 2002). If aging or lifespan is dictated by the presence of soluble toxic proteins, then reducing these toxic proteins would increase lifespan. As over‐expression of stv is unable to extend lifespan, this would then suggest that stv over‐expression is not sufficient to reduce these particular soluble toxic proteins, or that they are not targets of stv.

We find that Lamin, which is mutated to an aggregation prone protein form in Hutchinson‐Gilford progeria (Barascu et al., 2012; Burke & Stewart, 2013; Cao et al., 2007; Chandran et al., 2019; Eriksson et al., 2003), is a target of p38Kb and stv. Interestingly, p38Kb and Lamin mutants share similar phenotypes such as age‐dependent locomotor impairment and upregulation of the Nrf‐2/Keap‐1 pathway (Dialynas et al., 2015; Vrailas‐Mortimer et al., 2011). In addition, we find that Lam Dm_0_ localization is perturbed in p38Kb mutants, leading to chromosomal leakage from the nucleus, and the combined inhibition of p38Kb and Lam Dm_0_ is lethal. As loss of p38Kb leads to an accumulation of Lamin in the aggregates, this may be toxic to the cell, leading to impaired locomotor function, increased stress and decreased lifespan. Thus, we have found that one aging gene (p38Kb) regulates a second, unrelated aging gene (Lamin) via stv. These data suggest a new link between aging pathways and how they may converge through the regulation of protein homeostasis. As it has been recently reported that in mammals BAG‐3 can target a Lamin homologue for degradation (Gupta et al., 2019), the relationship between p38Kb, stv/BAG3, and Lamins may be conserved across species.

## 4 EXPERIMENTAL PROCEDURES

### 4.1 Genotypes

UAS‐p38Kb wt, UAS‐p38Kb Kinase Dead, p38Kb^Δ45^, p38Kb^Ex41^, w^1118^, Mef2‐GAL4 and MHC‐GAL4 were as described in (Vrailas‐Mortimer et al., 2011). The p38Kb^Ex41^ is a precise excision allele and serves as a genetic background control for p38Kb^Δ45^ deletion mutation.

UAS‐stv RNAi 34408 (w^1118^; P{GD10796}v34408) and UAS‐stv RNAi 34409 (w^1118^; P{GD10796}v34409/TM3) are described in (Dietzl et al., 2007) and are from the Vienna Drosophila Resource Center.

stv‐GFP trap (w^11118^;; Pbac{754.P.F3v30} stv^+CPTI 002824^), and HspB8‐GFP trap (w^1118^ PBac{810.P.FSVS‐2}CG14207^CPTI004445^) are from the Kyoto Stock Center.

w^1118^; P{y[+mDint2] w[BR.E.BR]=SUPor‐P}ref(2)P^KG00926^, w^1118^;;+mDint2 EY4969 stvEP, Lam^A25^ pr^1^, P{UAS‐mito‐HA‐GFP.AP}3, w[126]; P{w[+mC]=UAS‐Hsc70‐4.WT}B, w^1118^; P{w[+mC]=UAS‐Lam.GFP}3–3, y[1] v[1]; P{y[+t7.7] v[+t1.8]=TRiP.JF01389}attP2, and y[1] sc[*] v[1]; P{y[+t7.7] v[+t1.8]=TRiP.GL00577}attP2 were obtained from the Bloomington Drosophila Stock Center.

All fly stocks were backcrossed into the w^1118^ background and isogenized for 10 generations. All stocks were reared at 25°C in a 12 h:12 h light:dark cycle on standard fly food media. Virgin females were used for all experiments as they are larger than males, ensuring that sufficient muscle tissue could be obtained for data analysis.

### 4.2 Generation of UAS‐stv‐RA

The wildtype sequence of stv‐RA was cloned into pUAST‐attB (#1419 DGRC) by Genewiz and then injected into wild‐type embryos that have a PhiC31 integrase site C31 at 86F8 by BestGene, Inc.

### 4.3 Immunofluorescence

Adult virgin female flies were fixed in 4% paraformaldehyde for 48 h at 4°C. Indirect flight muscles were dissected in 1X PBS, permeabilized in 1X PBS 0.15% Triton‐X 100, and blocked in NGS +0.15% Triton‐X 100. Samples were incubated in primary antibody at 4°C overnight, washed in 1X PBS 0.15% Triton‐X 100, and incubated in secondary antibody at room temperature for 2 h. Samples were mounted in Vectashield mounting medium (Vectorlabs) and visualized using a Leica SP8 laser scanning confocal microscope. Antibodies: rabbit anti‐GFP 1:400 (Invitrogen), mouse anti‐FLAG M2 1:1000 (Sigma), rabbit anti‐stv 1:1000 (gift of Jög Höhfeld), rat anti‐α actinin 1:100 (Abcam), rabbit anti‐ubiquitin linkage‐specific K63 1:200 (Abcam), mouse anti‐Lam Dm_0_ 1:100 (DSHB), IgG‐ Alexa Fluor 488 1:200 (Life Technologies), anti‐mouse IgG‐ Alexa Fluor 568 1:500 (Life Technologies), anti‐rabbit IgG‐ Alexa Fluor 488 1:500 (Life Technologies), syto‐24 1:10,000 (Molecular Probes) and Rhodamine Phalloidin 1:2000 (Molecular Probes).

### 4.4 Protein aggregate analysis

Indirect flight muscle was prepared as described above from nine individual virgin female flies per genotype per age. Protein aggregates were identified using mouse anti‐poly‐ubiquitin 1:1000 (Enzo Life Sciences). Three muscles from each individual fly were imaged as z‐series and flattened into a single image as a max projection using confocal microscopy for a total of 27 muscles per genotype. Images were analyzed using Image J “Analyze Particles” function with a diameter of 100 pixels set for the minimum aggregate size. Aggregate number and size were analyzed using ANOVA followed by Tukey's HSD using the R (R Core Team R, 2021) package “multcomp” (Hothorn et al., 2008) to generate significance groups with each letter group being significantly different with a *p*‐value of ≤0.05. Within genotype/across time point analyses were performed using the Welch two sample *t*‐test in R.

### 4.5 Lifespan

For lifespan experiments using the UAS‐p38Kb^wt^, ref(2)p ‐/+, p38Kb^Δ45/Δ45^, the stv EP (stv^wt^), UAS‐Hsc70‐4 lines, and their respective controls, virgin females were kept on standard molasses *Drosophila* media made in the Emory Department of Cell Biology Fly Food Facility (Figures 2i, 4e, and Figure S5 and Tables S6, S10–S14). Due to a change in lab food, the stv RNAi 34408 and stv RNAi 34409 lifespan experiments (with their respective controls) were performed on the standard Bloomington *Drosophila* media (Figure 4b,c and Tables S10,S11), while the lifespan experiments for the stv EP expressed in the p38Kb^Δ45/Δ45^ mutant background (including all controls) were performed on standard molasses food (Genesee Scientific, Figure 4e and Table S13). Lifespan experiments were performed as described in (Vrailas‐Mortimer et al., 2011). Briefly, virgin females were collected and reared at 25°C in a 12 h:12 h light:dark cycle in groups of ~10–20 flies per vial and at least 10 replicate vials per genotype with a minimum of 50 flies total per genotype (See Supplemental Tables). Flies were put on new food twice a week or as needed. The number of dead animals was scored daily. Lifespan was analyzed using a log rank test to compare genotypes with censored data on all genotypes and then on all pairwise comparisons using the R package “survival” with Benjamini and Hochberg correction (false discovery rate <0.05).

### 4.6 Paraquat exposure

Virgin female flies were aged 1 week on standard fly food and then transferred to either standard fly food or standard fly food mixed with 20 mM paraquat (Sigma). Flies were collected when 25% of the control flies had died. Surviving flies were fixed in 4% paraformaldehyde and immunohistochemistry and protein aggregate analysis was performed as described above. 36 muscles per genotype were analyzed. Aggregate number was analyzed using ANOVA followed by Tukey's HSD using the R (R Core Team R, 2021) package “multcomp” (Hothorn et al., 2008) to generate significance groups with each letter group being significantly different with a *p*‐value of ≤0.05. In addition, single comparisons between control food and paraquat treated genotypes were analyzed using a Dunnett's test in R.

### 4.7 Flight assay

Virgin female flies were aged 1 week for interactions with ref(2)p (a minimum of 11 replicates per genotype) and 5 weeks for interactions with stv (a minimum of seven replicates per genotype) and then assayed for flight ability in the escape from free fall assay. Flies were introduced through a funnel into a 500 ml graduated cylinder coated with paraffin oil. The chamber is divided into thirds and the number of flies trapped in the paraffin oil in each third were tallied. Each replicate was performed with ~20 flies.

### 4.8 Co‐immunoprecipitation

Forty thoraxes per genotype per condition were homogenized in high salt buffer (0.5 M KCl, 35% glycerol,10 mM HEPES pH 7.0, 5 mM MgCl_2_, 0.5 mM EDTA pH 8.0, 0.1% NP40 25 mM NaF, 1 mM Na_2_VO_4_, 1 mM DTT, Complete protease inhibitor). The lysate was flash frozen in liquid nitrogen and quickly thawed at 37°C. Then lysates were rocked at 4°C for 30 min and centrifuged at 14,200 × g for 30 min at 4°C. The supernatant was transferred to equilibrated beads anti‐Flag (M2) agarose (Sigma) or anti‐GFP agarose (Chromotek) and rocked for 2 h at 4°C. Beads were collected using a magnetic bar and washed four times with IP buffer (50 mM HEPES pH 7.0, 100 mM KCl, 0.4% NP40 1.5 mM MgCl_2_, 5% glycerol, 25 mM Na, 1 mM Na_2_VO_4_, 1 mM EDTA, 1 mM DTT, Complete protease inhibitor). Lysates were then analyzed by immunoblotting using rabbit anti‐ GFP 1:1000 (Invitrogen), mouse anti‐FLAG M2 1:1000 (Sigma), rabbit total‐p38 1:1000 (Cell Signaling Technologies), rabbit anti‐phospho‐p38 1:1000 (Cell Signaling Technologies), goat anti‐total p38 1:1000 (Santa Cruz Biotechnology), rabbit anti‐stv 1:10,000 (gift of Jrög Höhfeld) or mouse anti‐Lamin 1:1000 (DHSB).

### 4.9 Immunoblotting

Wild‐type virgin female flies (w^1118^) were aged either for 3, 15, 30, and 45 days or for 1–5 weeks as indicated in Results. Three thoraxes were dissected and homogenized in 1x Laemmli buffer. Immunoblots were performed as described in (Vrailas‐Mortimer et al., 2011). Membranes were developed using SuperSignal West Femto kit (ThermoFisher) or Pierce ECL (ThermoFisher) and exposed on autoradiography film. Antibodies used were: rabbit anti‐GFP 1:1000 (Invitrogen), rabbit anti‐starvin 1:10,000 (gift of Jrög Höhfeld), mouse anti‐actin 1:5,000,000 (Sigma), mouse anti‐FLAG M2 (Sigma), rabbit anti‐alpha tubulin (Cell Signaling Technologies), mouse anti‐Lamin 1:100 (DHSB), rabbit anti‐phospho‐Lamin A Ser22 1:1000 (Thermofisher), mouse anti‐beta tubulin (E‐10) 1:5000 (Santa Cruz Biotechnology), mouse anti‐ HRP 1:20,000 (Jackson Labs), rabbit anti‐HRP 1:40,000 (Jackson Labs). Densitometry was performed using a minimum of three independent blots. For statistical analysis of protein expression level, pixel density of the tested protein was normalized within sample to the loading control. These values were then normalized to control to calculate fold change. The fold change values were analyzed by Student's *t*‐test or ANOVA (R. C. Team, 2015) as appropriate.

### 4.10 Sucrose gradient fractionation

p38Kb^Ex41/Ex41^ and p38Kb^Δ45/Δ45^ virgin female flies were aged three weeks. 30 thoraxes per genotype were dissected and homogenized in NP40 lysis buffer. Samples were centrifuged at 800 × g for 10 min at 4°C to remove organelles and cellular membranes. The supernatant was then transferred to a 15%–50% sucrose discontinuous gradient. Samples were then ultracentrifuged at 259,000x *g* for 20 h at 4°C in a TLS‐55 in a Beckman Coulter Optima TLX Ultracentrifuge. 200 µl fractions were collected, and the pellet was resuspended in an equal volume of NP40 Lysis Buffer.

### 4.11 Stv localization

Immunohistochemistry on p38Kb^Ex41/ Ex41^ and p38Kb^Δ45/Δ45^ Indirect flight muscles was performed as described above. Confocal images from five individual virgin female flies per genotype were analyzed for average pixel density using ImageJ in three different non‐overlapping locations on each muscle for a total of 15 measurements per genotype. Average pixel density was analyzed by Student's *t*‐test using R.

## CONFLICT OF INTEREST

The authors have no conflicts of interest.

## AUTHOR CONTRIBUTIONS

S. Ryan, M. Almassey, A. Burch, G. Ngo, J. Martin, D. Myers, D. Compton, S. Archie, M. Cross, L. Naeger, A. Salzman, A. Virola‐Iarussi, N. Mortimer, and A. Vrailas‐Mortimer performed experiments. S. Ryan, N. Mortimer, S. Barbee, S. Sanyal, and A. Vrailas‐Mortimer made intellectual contributions to experimental design and data interpretation. All authors were involved in reading and editing the manuscript.

## Supporting information

Fig S1

Fig S2

Fig S3

Fig S4

Fig S5

Fig S6

Fig S7

Table S1‐S17

Supplementary Material

## Data Availability

The data that support the findings of this study are available from the corresponding author upon reasonable request.
