# Supplementary figures and images for: Drosophila p38 MAPK interacts with BAG‐3/starvin to regulate age‐dependent protein homeostasis
Source: Aging Cell. 2021 Oct 21;20(11):e13481. doi: 10.1111/acel.13481 (PMC8590102; doi:10.1111/acel.13481)

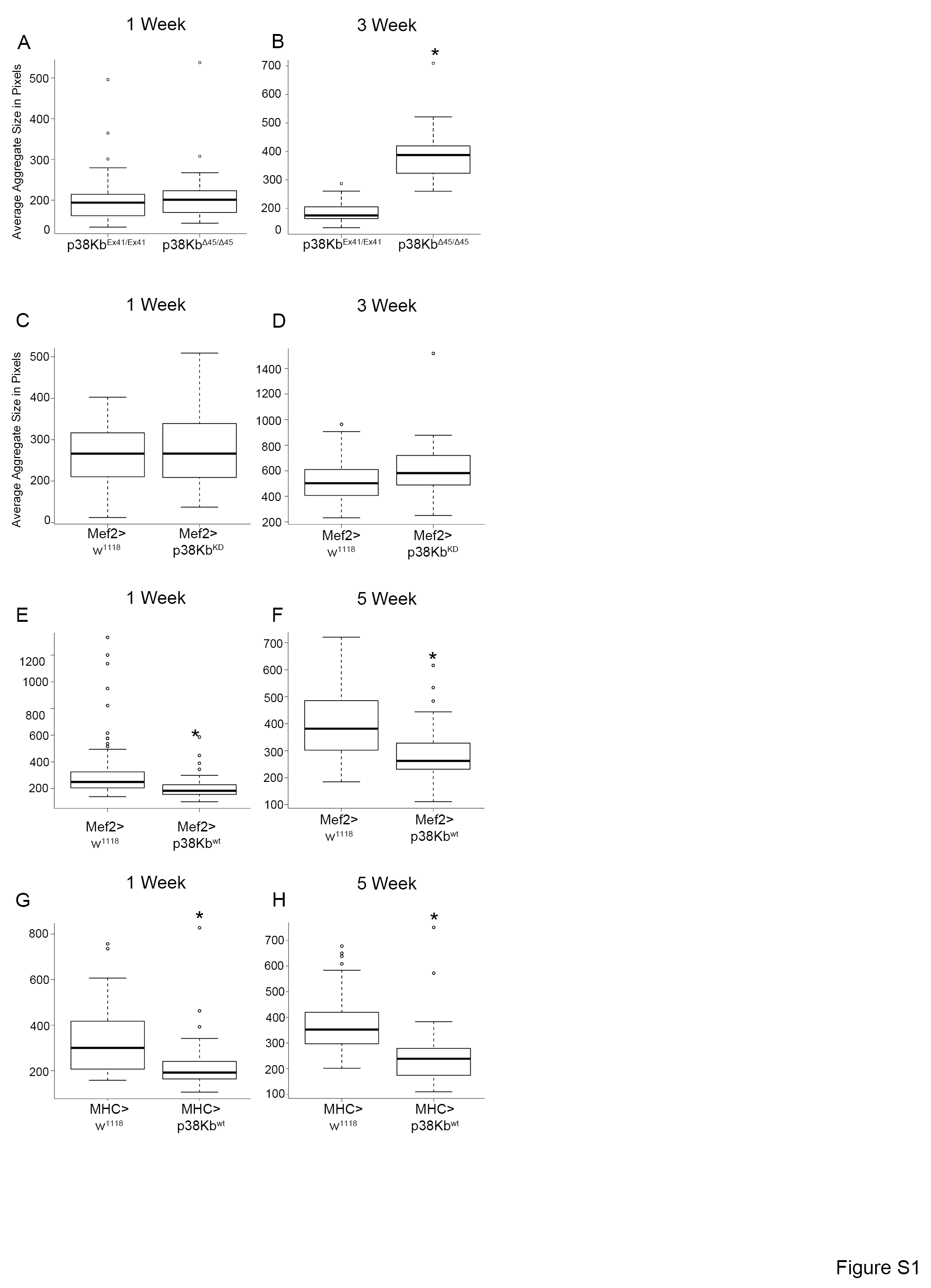

Supplement: Supplementary file 1 — Fig S1 [file ACEL-20-e13481-s006.tif]

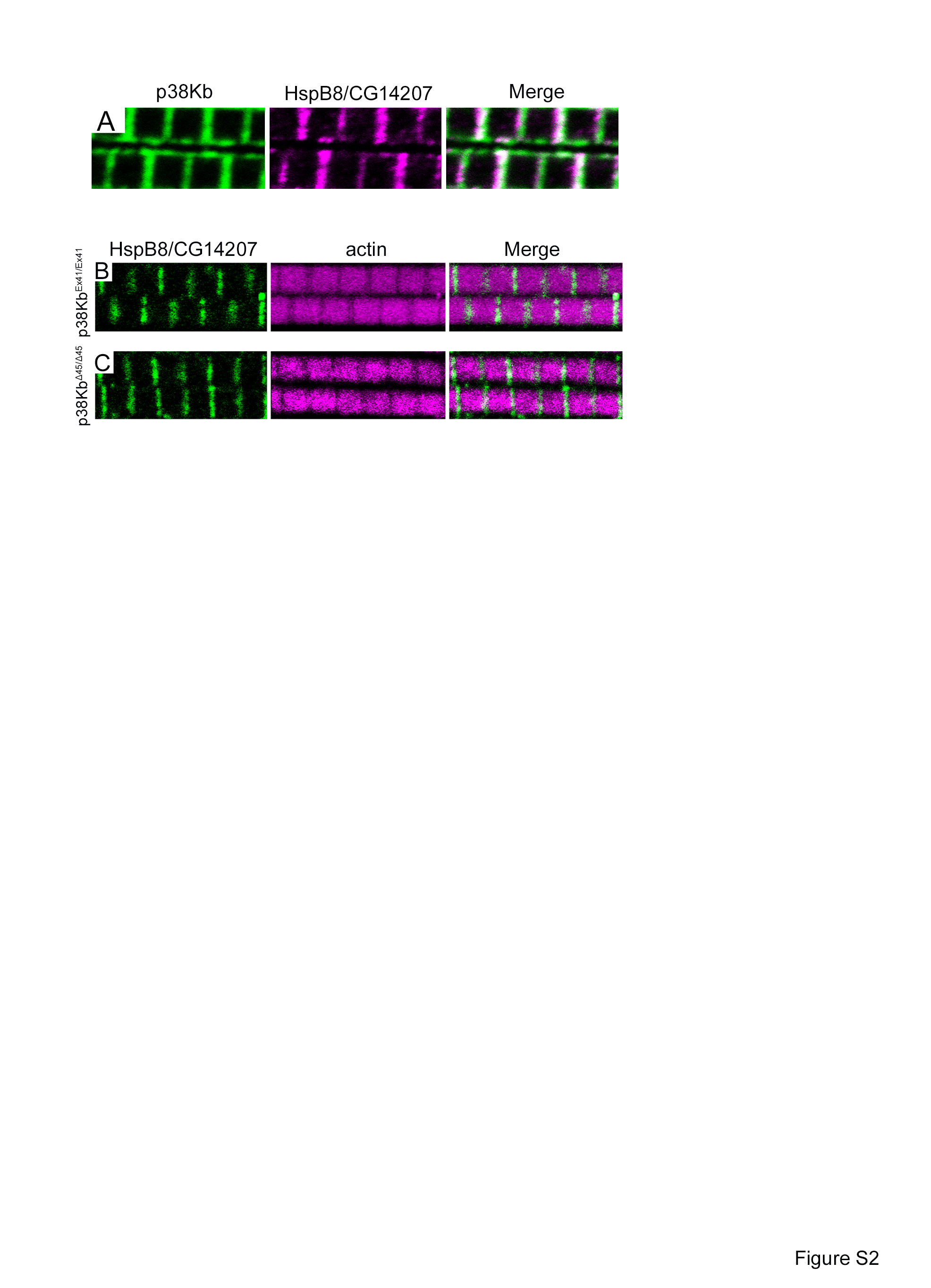

Supplement: Supplementary file 2 — Fig S2 [file ACEL-20-e13481-s007.tif]

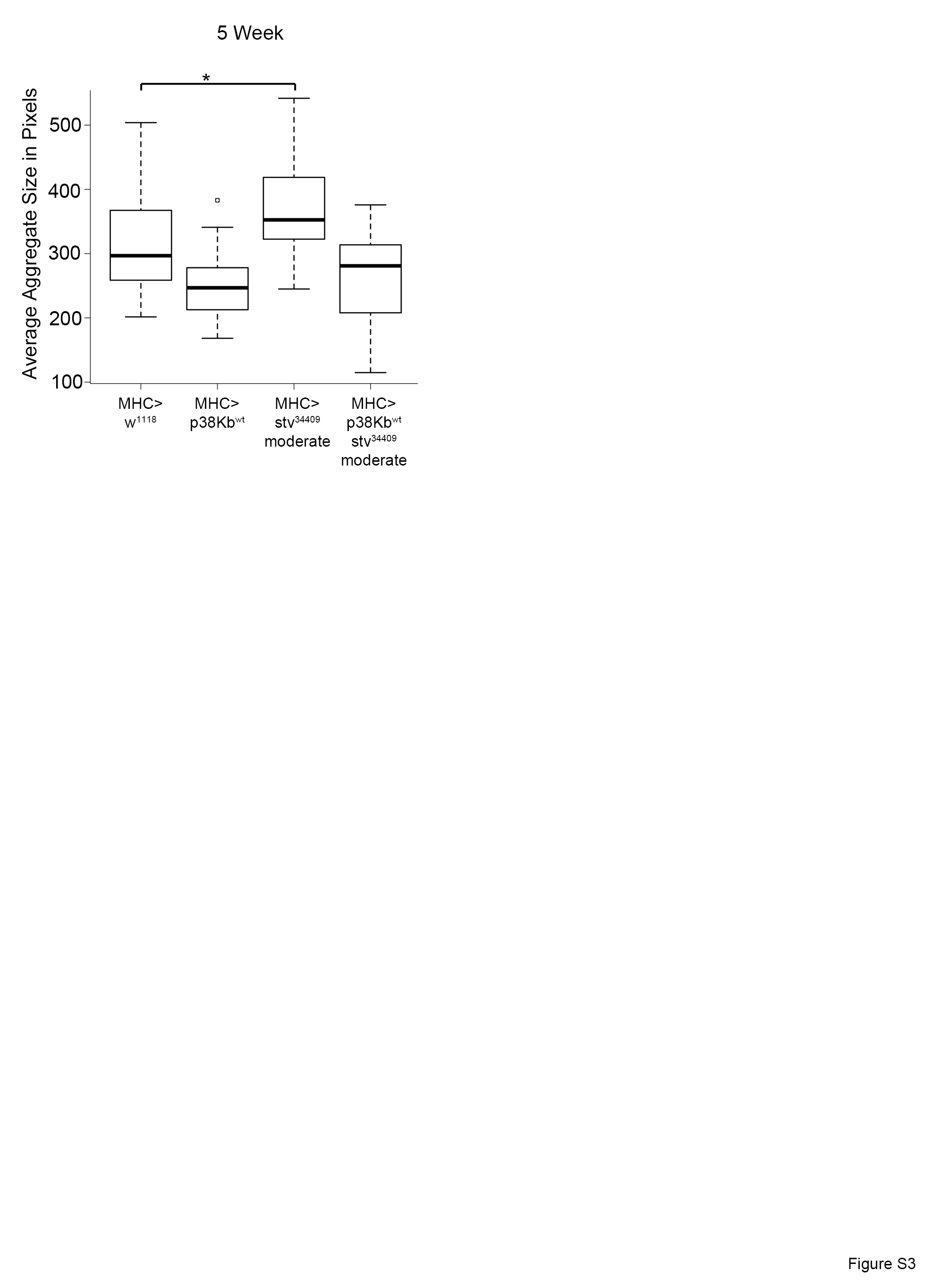

Supplement: Supplementary file 3 — Fig S3 [file ACEL-20-e13481-s005.tif]

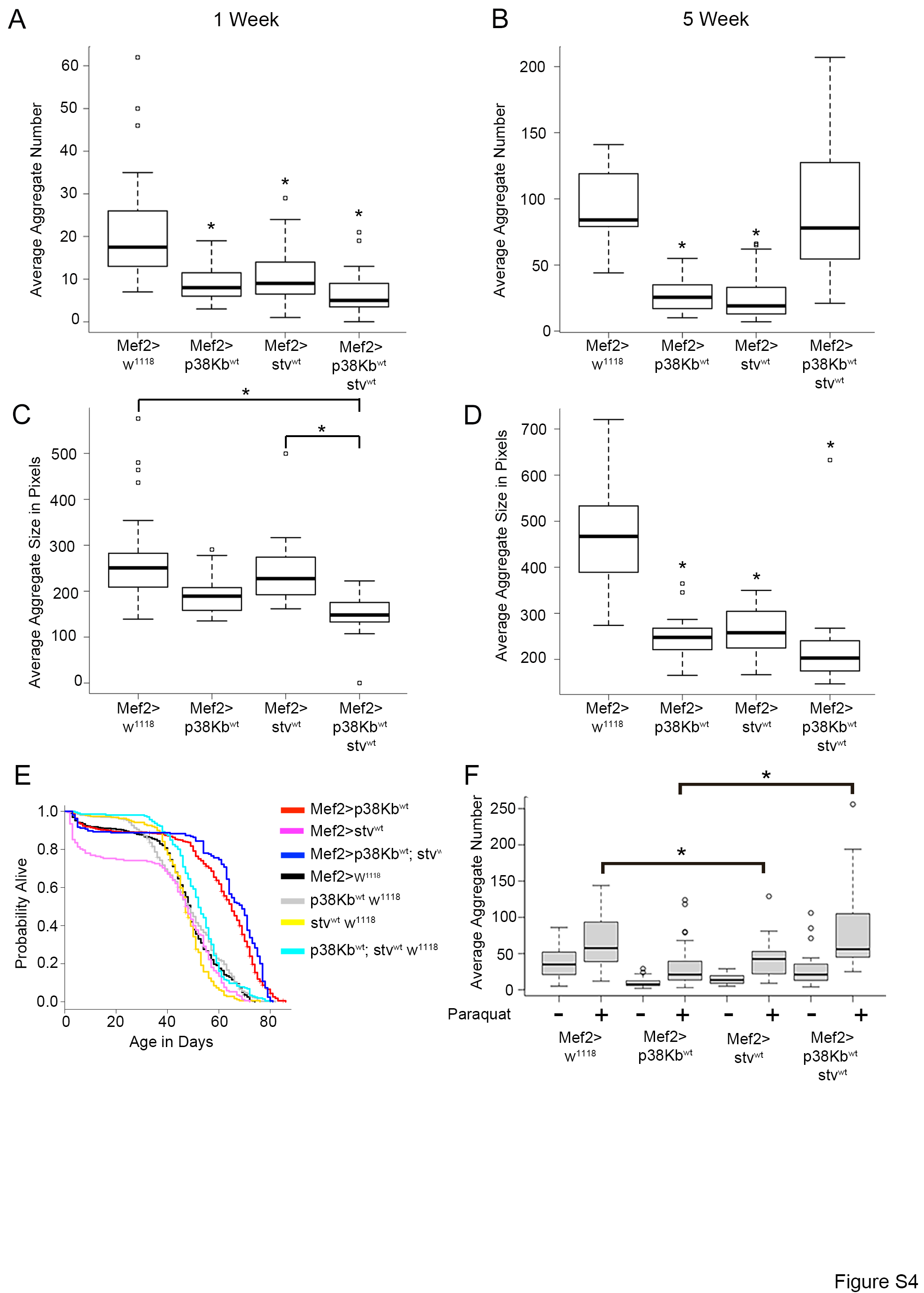

Supplement: Supplementary file 4 — Fig S4 [file ACEL-20-e13481-s002.tif]

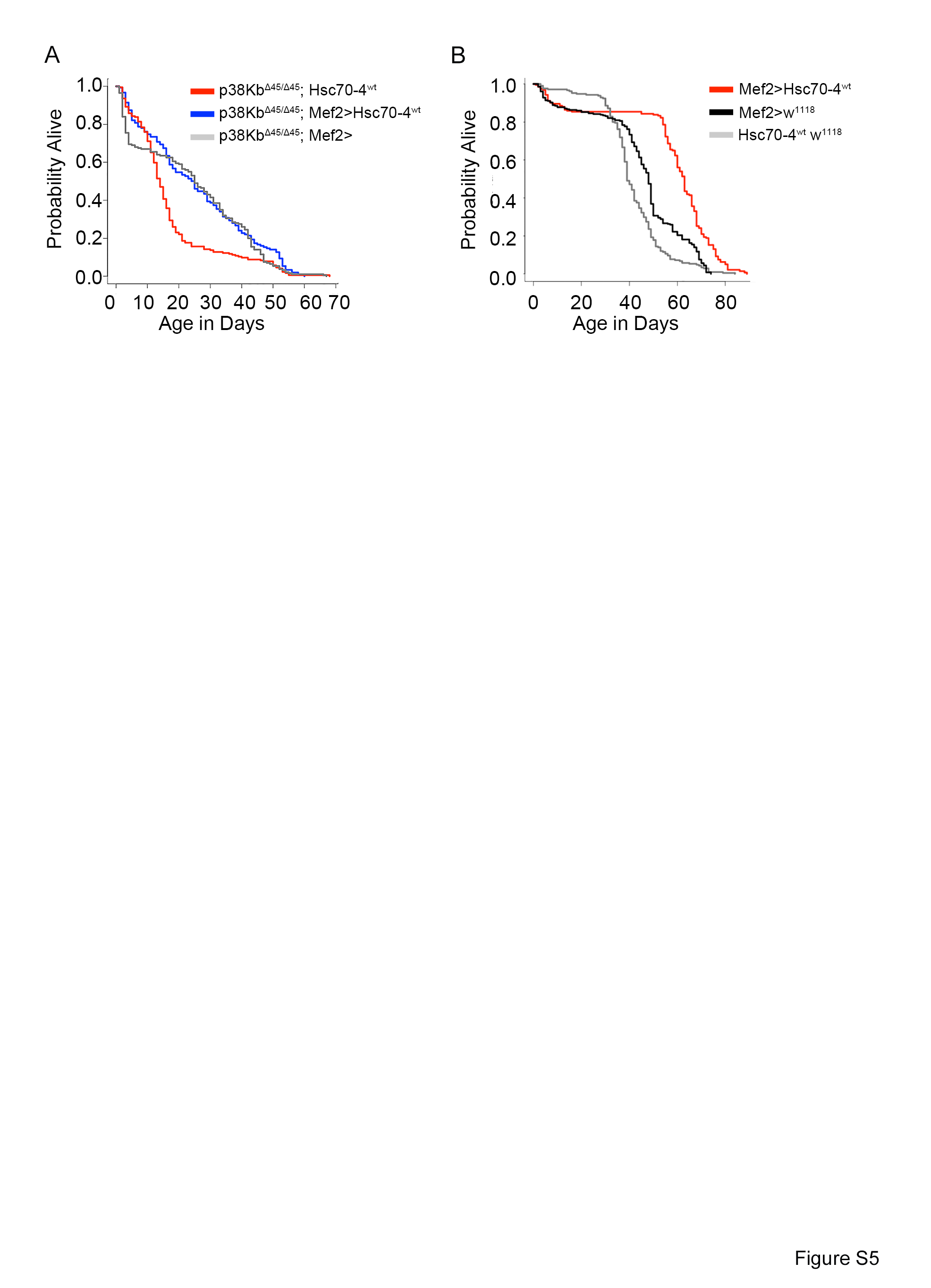

Supplement: Supplementary file 5 — Fig S5 [file ACEL-20-e13481-s004.tif]

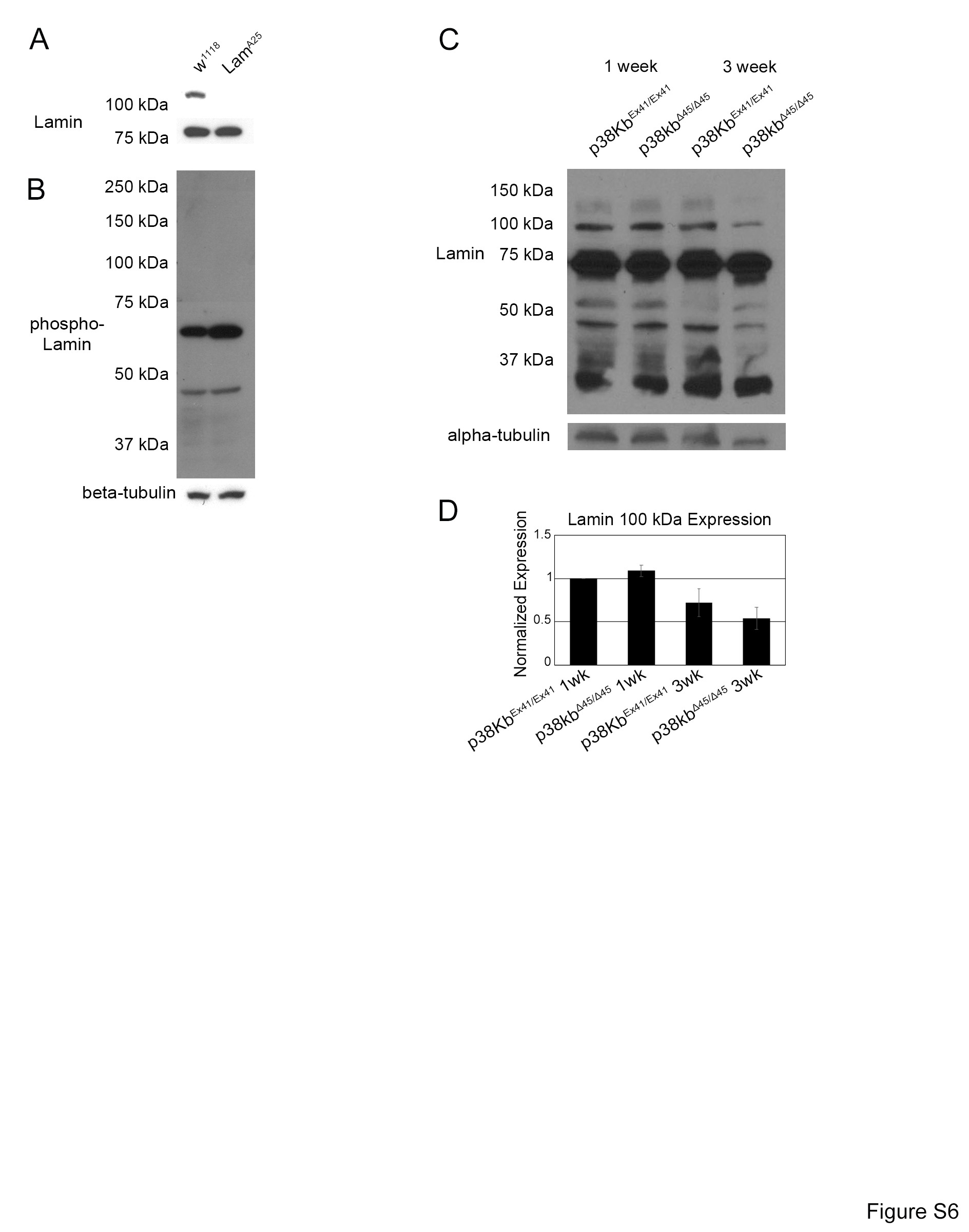

Supplement: Supplementary file 6 — Fig S6 [file ACEL-20-e13481-s001.tif]

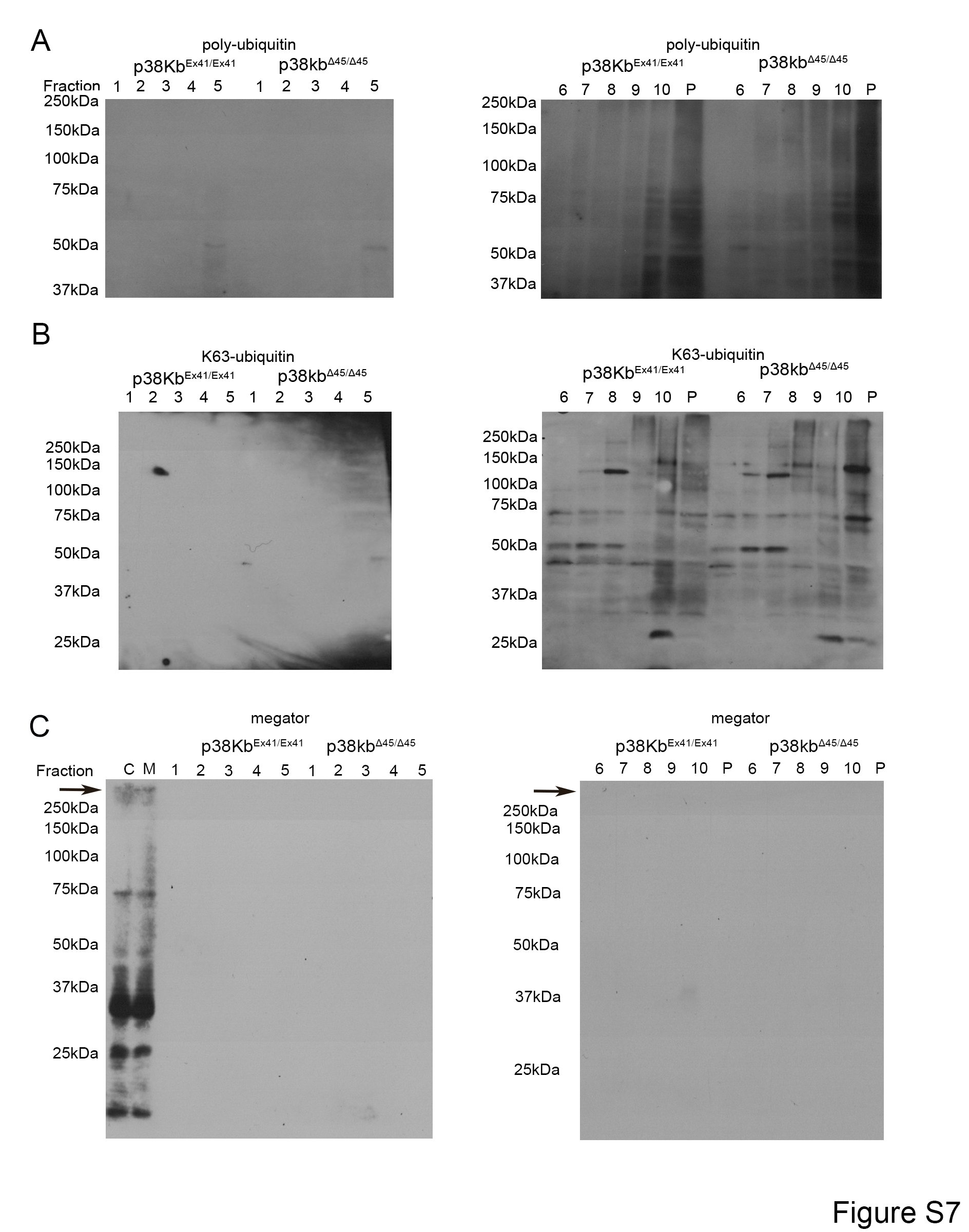

Supplement: Supplementary file 7 — Fig S7 [file ACEL-20-e13481-s003.tif]
