# Supplementary material for: Drosophila p38 MAPK interacts with BAG‐3/starvin to regulate age‐dependent protein homeostasis
Source: Aging Cell. 2021 Oct 21;20(11):e13481. doi: 10.1111/acel.13481 (PMC8590102; doi:10.1111/acel.13481)
Supplement: Supplementary file 8 — Table S1‐S17 [file ACEL-20-e13481-s008.pdf]

Genotype	Aggregate Number 1 Week	Aggregate Number 3 Week	Aggregate Size 1 Week	Aggregate Size 3 Week
p38Kb $^{Ex41/Ex41}$	8.8 ± 0.99	45.6 ± 5.09	209.8 ± 14.79	190.7 ± 8.15
p38Kb $^{\Delta 45/\Delta 45}$	38.5 ± 4.28*	139.19 ± 10.11*	214.2 ± 14.38	385.2 ± 18.14*

Table S1. Loss of p38Kb affects aggregate number and size. Mean aggregate number and size at 1 and 3 weeks. Asterisks indicate a p value of < 0.001.

Genotype	Aggregate Number 1 Week	Aggregate Number 5 Week	Aggregate Size 1 Week	Aggregate Size 5 Week
Mef2>w^{1118}	6.1 ± 1.18	57.6 ± 6.27	264.9 ± 21.09	540.09 ± 39.76
Mef2> $p38Kb^{KD}$	16.7 ± 3.94*	91.3 ± 11.67*	277.3 ± 23.30	631.7 ± 48.92

Table S2. Expression of a dominant negative p38Kb Kinase Dead affects aggregate number. Mean aggregate number and size at 1 and 5 weeks. Asterisks indicate a p value of <0.02.

Genotype	Aggregate Number 1 Week	Aggregate Number 5 Week	Aggregate Size 1 Week	Aggregate Size 5 Week
Mef2>w^{1118}	24.5 ± 2.19	92.0 ± 3.72	310.3 ± 19.91	398.8 ± 13.69
Mef2>$p38Kb^{wt}$	6.7 ± 0.47*	37.7 ± 2.35*	199.2 ± 6.81*	287.1 ± 10.03*

Table S3. Strong over-expression of p38Kb affects aggregate number and size. Mean aggregate number and size at 1 and 5 weeks. Asterisks indicate a p value of < 0.001.

Genotype	Aggregate Number 1 Week	Aggregate Number 5 Week	Aggregate Size 1 Week	Aggregate Size 5 Week
MHC>w^{1118}	18.0 ± 2.02	63.9 ± 5.83	332.3 ± 19.02	375.2 ± 15.74
MHC>$p38Kb^{wt}$	5.8 ± 0.59*	17.5 ± 1.52*	219.8 ± 15.6*	244.4 ± 14.40*

Table S4. Moderate over-expression of p38Kb affects aggregate number and size. Mean aggregate number and size at 1 and 5 weeks. Asterisks indicate a p value of < 0.001.

Genotype	Aggregate Number 1 Week	Aggregate Number 5 Week	Aggregate Size 1 Week	Aggregate Size 5 Week
Mef2>w1118	12.6 ± 1.01 b	100.0 ± 6.55 c	248.0 ± 24.77 b	334.1 ± 13.43 ab
Mef2>p38Kb^{wt}	4.0 ± 0.56 a	34.9 ± 2.59 a	174.8 ± 9.01 a	381.1 ± 16.68 b
Mef2>ref(2)p$^{-/+}$	7.6 ± 1.15 ab	74.2 ± 5.69 b	181.6 ± 11.79 a	324.1 ± 13.66 a
Mef2>p38Kb^{wt} ref(2)p-/+	19.6 ± 2.46 c	108.1 ± 7.83 c	185.6 ± 5.93 a	339.8 ± 13.86 ab

Table S5. Reduction of ref(2)p affects p38Kb aggregate number. Mean aggregate number and size at 1 and 5 weeks. Means not sharing the same letter (a, b, c) are significantly different (Tukey's HSD, $p<0.05$).

Genotype	Average Age	Median Age	n	p value vs GAL4 Control	p value vs UAS-p38Kb^{wt} Mef2-GAL4
Mef2>w1118	46.3 days	49 days	515	-	0
Mef2>p38Kb^{wt}	58.2 days	64 days	730	0	-
ref(2)p -/+ Mef2-GAL4	42.3 days	52 days	187	0.829	0
UAS-p38Kb^{wt}; ref(2)p -/+ Mef2-GAL4	48.7 days	57 days	190	2.97E-05	0
UAS-p38Kb^{wt} W1118	47.4 days	48 days	598	0.1901053	0
ref(2)p -/+ W1118	43 days	46 days	185	6.03E-04	0
UAS-p38Kb^{wt}; ref(2)p -/+ W1118	35.5 days	34 days	193	0	0

Table S6. Inhibition of *ref(2)p* prevents p38Kb lifespan extension. Log rank Chisq = 750, p = 0.

	Aggregate Number - PQ	Aggregate Number + PQ	p value -PQ vs +PQ
W1118 Mef2-GAL4	29.8 ± 3.28 b	43.4 ± 4.46 b	0.01637
p38Kb wt Mef2-GAL4	8.1 ± 1.03 a	11.7 ± 1.33 a	0.03910
ref(2)p-/+ Mef2-GAL4	24.5 ± 2.77 b	47.8 ± 6.31 b	0.00144
p38Kb wt ref(2)p-/+ Mef2-GAL4	41.8 ± 4.54 c	76.9 ± 8.11 c	0.00039

Table S7. Reduction of *ref(2)p* affects p38Kb aggregate number. Mean aggregate number on control food or paraquat food. Means not sharing the same letter (a, b, c) are significantly different (Tukey's HSD, $p<0.05$) within that condition.

Genotype	Aggregate Number 1 Week	Aggregate Number 5 Week	Aggregate Size 1 Week	Aggregate Size 5 Week
MHC>w^{1118}	12.5 ± 1.21 b	93.2 ± 7.43 b	309.9 ± 19.15 a	437.3 ± 22.23 c
MHC>$p38Kb^{wt}$	5.1 ± 0.48 a	19.4 ± 2.81 a	228.1 ± 24.89 a	236.6 ± 27.39 a
MHC>stv^{34408} RNAi	11.4 ± 1.35 b	72.1 ± 9.05 b	293.3 ± 30.79 a	362.0 ± 9.66 b
MHC>$p38Kb^{wt}$ stv^{34408} RNAi	14.1 ± 1.84 b	29.8 ± 4.23 a	233.9 ± 11.93 a	261.7 ± 12.84 a

Table S8. Mild inhibition of stv has no effect on p38Kb aggregate number or size. Mean aggregate number and size at 1 and 5 weeks. Means not sharing the same letter (a, b, c) are significantly different (Tukey's HSD, $p<0.05$).

Genotype	Aggregate Number 1 Week	Aggregate Number 5 Week	Aggregate Size 1 Week	Aggregate Size 5 Week
MHC>w^{1118}	23.6 ± 3.58 bc	33.6 ± 3.87 b	354.6 ± 33.15 b	310.8 ± 14.25 b
MHC>$p38Kb^{wt}$	6.4 ± 1.08 a	15.6 ± 1.20 a	210.5 ± 17.22 a	252.2 ± 10.32 a
MHC>stv^{34409} RNAi	28.2 ± 6.40 c	60.4 ± 6.99 c	234.1 ± 15.28 a	363.1 ± 13.67 c
MHC>$p38Kb^{wt}$ stv^{34409} RNAi	9.8 ± 1.75 ab	35.5 ± 4.90 b	281.7 ± 40.04 ab	263.9 ± 13.62 ab

Table S9. Moderate inhibition of stv has affects p38Kb aggregate number but not size. Mean aggregate number and size at 1 and 5 weeks. Means not sharing the same letter (a, b, c) are significantly different (Tukey's HSD, $p<0.05$).

Genotype	Average Age	Median Age	n	p value vs MHC>stv^{34409} RNAi	p value vs MHC>p38Kb^{wt}	p value vs MHC>w^{1118}	p value vs Transgene Control
MHC>w^{1118}	51 days	52 days	212	4.00E-03	0	-	-
MHC>p38Kb^{wt}	73.5 days	77 days	175	0	-	0	6.48E-13
MHC>stv^{34409} RNAi	44.3 days	48 days	217	-	0	4.00E-03	3.32E-06
MHC>p38Kb^{wt} stv^{34409} RNAi	56.9 days	60.5 days	182	1.49E-04	0	3.54E-12	0
p38Kb^{wt} w^{1118}	59.4 days	70 days	178	1.93E-09	6.48E-13	0	-
stv^{34409} w^{1118}	46.2 days	50.5 days	194	3.31E-06	0	7.78E-06	-
p38Kb^{wt} stv^{34409} RNAi w^{1118}	41.3 days	41 days	197	7.68E-10	0	0	-

Table S10. Moderate inhibition of stv prevents p38Kb lifespan extension. Chisq = 551, p = 0.

Genotype	Average Age	Median Age	n	p value vs Mef2>stv^{34408}	p value vs Mef2>p38Kb^{wt}	p value vs Mef2>w^{1118}	p value vs Transgene Control
Mef2>w1118	38 days	42 days	206	0	0	-	-
Mef2>p38Kb^{wt}	74.2 days	79 days	203	0	-	0	0
Mef2>stv^{34408} RNAi	4.2 days	4 days	116	-	0	0	0
Mef2>p38Kb^{wt} stv^{34408} RNAi	13.4 days	4 days	208	1.86E-07	0	1.33E-10	0
p38Kb^{wt} w^{1118}	52.2 days	52 days	203	0	0	0	-
stv^{34408} RNAi w^{1118}	65.8 days	67 days	206	0	0	0	-
p38Kb^{wt} stv^{34408} RNAi w^{1118}	66.3 days	68 days	203	0	6.48E-16	0	-

Table S11. Strong inhibition of stv prevents p38Kb lifespan extension. Chisq = 1693, p = 0.

Genotype	Average Age	Median Age	n	p value vs Mef2>w^{1118}	p value vs. Mef2>p38Kb$^{\Delta45/\Delta45}$	p value vs. Transgene Control
On Molasses Food (hand made)						
Mef2>p38Kb$^{\Delta45/\Delta45}$	24.1 days	25 days	200	-	-	-
p38Kb$^{\Delta45/\Delta45}$ stv^{wt}	25.6 days	25 days	207	-	0.936	-
Mef2>p38Kb$^{\Delta45/\Delta45}$; stv^{wt}	15.5 days	10 days	50	-	1.03E-04	1.87E-06
On Molasses Food (Genesee Scientific)						
Mef2>w^{1118}	74.3 days	78 days	464	-	2.00E-16	2.00E-16
Mef2>p38Kb$^{\Delta45/\Delta45}$	43.1 days	47 days	388	2.00E-16	-	2.50E-05
p38Kb$^{\Delta45/\Delta45}$ stv^{wt}	39.6 days	45 days	406	2.00E-16	2.50E-05	-
Mef2>p38Kb$^{\Delta45/\Delta45}$; stv^{wt}	26.7 days	25.5 days	196	2.00E-16	2.00E-16	5.10E-16

Table S12. Over-expression of stv fails to rescue p38Kb mutant short lifespan. Molasses Food handmade Chisq = 22.7, p = 1.2E-05. Molasses Food from Genesee Scientific Chisq = 1133, p <2E-16.

Genotype	Average Age	Median Age	n	p value vs Mef2>stv^{wt}	p value vs Mef2>p38Kb^{wt}	p value vs Mef2>w^{1118}	p value vs Transgene Control
Mef2>w^{1118}	46.3 days	49 days	515	0.03458824	0	-	-
Mef2>p38Kb^{wt}	58.2 days	64 days	730	0	-	0	0
Mef2>stv^{wt}	39.7 days	47 days	182	-	0	0.034588	0.195
Mef2>p38Kb^{wt} stv^{wt}	61.3 days	69 days	205	0	0.03651667	0	0
p38Kb^{wt} w^{1118}	47.4 days	48 days	598	3.51E-04	0	0.1806	-
stv^{wt} w^{1118}	46.1 days	47 days	189	0.195	0	3.47E-03	-
p38Kb^{wt} stv^{wt} w^{1118}	51.9 days	52 days	206	1.17E-04	0	2.00E-03	-

Table S13. p38Kb and stv co-over-expression further extends lifespan. Chisq = 706, p = 0.

Genotype	Average Age	Median Age	n	p value vs GAL4 Control	p value vs transgene control
Mef2>p38Kb$^{\Delta45/\Delta45}$	24.14 days	25 days	200	-	0.00025
p38Kb$^{\Delta45/\Delta45}$ Hsc70-4^{wt}	19.80 days	15 days	172	0.00025	-
Mef2>p38Kb$^{\Delta45/\Delta45}$; Hsc70-4^{wt}	25.30 days	24 days	152	0.32214	9.50E-06

Table S14. Over-expression of Hsc70-4 fails to rescue p38Kb mutant short lifespan. Chisq= 25.1, p= 4e-06.

Genotype	Average Age	Median Age	n	p value vs Mef2>w^{1118}	p value vs Transgene Control
Mef2>w1118	44.5 days	49 days	278	-	2.10E-06
Hsc70-4^{wt} w^{1118}	41.4 days	39 days	210	2.10E-06	-
Mef2>Hsc70-4^{wt}	56.32 days	63 days	192	< 2e-16	< 2e-16

Table S15. Hsc70-4 over-expression further extends lifespan. Chisq = 149, p < 2e-16.

Genotype	Aggregate Number 1 Week	Aggregate Number 5 Week	Aggregate Size 1 Week	Aggregate Size 5 Week
Mef2>w1118	21.8 ± 2.64 b	92.7 ± 5.10 b	276.1 ± 21.07 c	472.0 ± 20.95 b
Mef2>p38Kb^{wt}	8.7 ± 0.77 a	27.5 ± 2.41 a	192.2 ± 7.87 ab	249.0 ± 8.11 a
Mef2>stv^{wt}	10.3 ± 1.22 a	25.7 ± 3.39 a	242.0 ± 13.51 bc	258.6 ± 9.45 a
Mef2>p38Kb^{wt} stv^{wt}	6.8 ± 1.01 a	92.5 ± 9.48 b	153.3 ± 5.68 a	221.3 ± 17.29 a

Table S16. Over-expression of stv affects p38Kb aggregate size at a young age. Mean aggregate number and size at 1 and 5 weeks. Means not sharing the same letter (a, b, c) are significantly different (Tukey's HSD, $p<0.05$).

Genotype	Aggregate Number 1 Week	Aggregate Number 5 Week	Aggregate Size 1 Week	Aggregate Size 5 Week
Mef2>w1118	21.8 ± 2.64 b	92.7 ± 5.10 b	276.1 ± 21.07 c	472.0 ± 20.95 b
Mef2>p38Kb^{wt}	8.7 ± 0.77 a	27.5 ± 2.41 a	192.2 ± 7.87 ab	249.0 ± 8.11 a
Mef2>stv^{wt}	10.3 ± 1.22 a	25.7 ± 3.39 a	242.0 ± 13.51 bc	258.6 ± 9.45 a
Mef2>p38Kb^{wt} stv^{wt}	6.8 ± 1.01 a	92.5 ± 9.48 b	153.3 ± 5.68 a	221.3 ± 17.29 a

Table S17. p38Kb does not interact with stv to regulate protein homeostasis in response to oxidative stress. Mean aggregate number on control food or paraquat food. Means not sharing the same letter (a, b, c) are significantly different (Tukey's HSD, p<0.05) within that condition.
