# Supplementary material for: Drosophila p38 MAPK interacts with BAG‐3/starvin to regulate age‐dependent protein homeostasis
Source: Aging Cell. 2021 Oct 21;20(11):e13481. doi: 10.1111/acel.13481 (PMC8590102; doi:10.1111/acel.13481)
Supplement: Supplementary file 9 — Supplementary Material [file ACEL-20-e13481-s009.pdf]

Supplemental Figure Legends

Fig S1. p38Kb regulates age-dependent protein homeostasis.

Box-Whisker plots of aggregate size in p38Kb $^{\Delta45/\Delta45}$ mutants as compared to p38Kb$^{Ex41/Ex41}$ controls at **A)** 1 week and **B)** 3 weeks of age. Aggregate size in p38Kb^{KD} Mef2-GAL4 (Mef2>p38Kb^{KD}) and outcrossed Mef2-GAL4 controls at **C)** 1 week and **D)** 5 weeks of age. Aggregate size in strong p38Kb over-expression animals and outcrossed Mef2-GAL4 controls at **E)** 1 week and **F)** 5 weeks of age. Aggregate size in moderate p38Kb over-expression (MHC>p38Kb^{wt}) animals and outcrossed MHC-GAL4 controls (MHC>w^{1118}) at **G)** 1 week and **H)** 5 weeks of age. Asterisks denote a p-value of ≤0.001.

Fig S2. Localization of HspB8 in p38Kb mutants. A) Endogenously GFP-tagged HspB8 colocalizes with p38Kb. **B-C)** GFP-tagged HspB8 localization was analyzed in the muscle of three week old **B)** controls and **C)** p38Kb mutants. HspB8 (green) localizes to the Z-disc and M-line of the indirect flight muscle (actin in magenta) in both control and p38Kb mutant animals.

Fig S3. p38Kb acts downstream of stv to regulate aggregate size. Box-Whisker plots of aggregate size in the moderate stv knockdown background using MHC-GAL4 at 5 weeks. Asterisks denote a p-value of ≤0.001. Inhibition of stv leads to increased aggregate size, which is rescued by over-expression of p38Kb.

Fig S4. p38Kb is limiting for stv function.

Protein aggregate number at **A)** 1 week and **B)** 5 weeks and protein aggregate size at **C)** 1 week and **D)** 5 weeks measured in stv over-expression backgrounds. Over-expression of stv leads to reduced aggregate number at 1 and 5 weeks and aggregate size at 5 weeks. Co-over-expression of stv and p38Kb does not result in a further

decrease in protein aggregate number but trends towards decreased aggregate size at both 1 and 5 weeks of age. Asterisks denote a p-value of ≤0.001 when compared to the GAL4 control. **E)** Over-expression of stv alone has no significant effect on lifespan (pink line as compared to yellow and black lines), however, co-over-expression of stv and p38Kb results in a further increase in lifespan (compare red line to blue line). **F)** Number of polyubiquitin positive protein aggregates in response to oxidative stress. Paraquat exposure leads to a significant increase in protein aggregation for all genotypes as compared to controls. Over-expression of stv is protective against oxidative stress but does not interact with p38Kb to further reduce paraquat induced protein aggregation. Asterisks denote a p-value of ≤0.05.

Fig S5. Over-expression of Hsc70-4 does not rescue p38Kb mutant shortened lifespan. A) Over-expression of wild type Hsc70-4 in the muscle of p38Kb mutants (blue line) is unable to rescue the p38Kb mutant short lifespan phenotype (red and grey lines). **B)** Muscle over-expression of wild type Hsc70-4 in a wild type background (red line) results in a lifespan extension as compared to controls (black and grey lines).

Fig S6. Expression of Lam Dm_0 in muscle.

A) Immunoblot of Lam Dm_0 in control and Lam Dm_0 heterozygous mutant that lacks the CAAX box. The 100kDa form of Lam Dm_0 is missing in the Lam Dm_0 mutants, suggesting that this is the farneslyated form. **B)** Phosphorylation of Lamin is not altered in the Lam Dm_0 mutants. **C)** Immunoblot analysis of p38Kb$^{Ex41/Ex41}$ control and p38Kb $^{\Delta45/\Delta45}$ mutant muscle lysates probed with anti-Lamin Dm_0 and **D)** quantification of the 100kDa form using densitometry. The 100kDa form of Lam Dm_0 is not as highly expressed as the main 75kDa form. Levels of the 100kDa form are not altered by loss of p38Kb.

Fig 7. Polyubiquitin positive protein aggregates are increased in aged p38Kb mutants.

Immunoblots of sucrose gradient fractions from 3 week old p38Kb$^{Ex41/Ex41}$ control and p38Kb $^{\Delta45/\Delta45}$ mutant muscle. **A)** Poly-ubiquitin is found mostly in the pellet fraction, marking the protein aggregates and is more prevalent in the pellet fraction of p38Kb mutants. **B)** Protein with K63 ubiquitination are present in fractions 5-10 and the pellet. p38Kb mutants have increased K63 ubiquitination in the pellet as compared to controls. **C)** Megator is a 250kDa member of the nuclear pore complex and is found in whole muscle lysate (lane C, p38Kb$^{Ex41/Ex41}$ and lane M, p38Kb $^{\Delta45/\Delta45}$) but not in the organelle free fractions or pellet.
